# “It Felt Good to Be Able to Say That Out Loud”—Therapeutic Alliance and Processes in AVATAR Therapy for People Who Hear Distressing Voices: Peer-Led Qualitative Study

**DOI:** 10.2196/77566

**Published:** 2026-01-28

**Authors:** Emily Rutter-Eley, Thomas Craig, Philippa Garety, Mar Rus-Calafell, Hannah Ball, Moya Clancy, Jeffrey McDonnell, Andrew Gumley, Gillian Haddock, Sandra Bucci, Miriam Fornells-Ambrojo, Nerys Baldwin, Jed Harling, Alie Phiri, Charlie MacKenzie-Nash, Nicholas Hamilton, Amy Grant, Clementine Edwards, Thomas Ward

**Affiliations:** 1 Department of Psychology Institute of Psychiatry, Psychology & Neuroscience King's College London London United Kingdom; 2 South London & Maudsley NHS Foundation Trust London United Kingdom; 3 Department of Health Service and Population Research Institute of Psychiatry, Psychology & Neuroscience King's College London London United Kingdom; 4 Mental Health Research and Treatment Center Faculty of Psychology Ruhr University Bochum Bochum Germany; 5 Division of Psychology and Mental Health School of Health Sciences University of Manchester and the Manchester Academic Health Sciences Centre Manchester United Kingdom; 6 Greater Manchester Mental Health NHS Foundation Trust and the Manchester Academic Health Sciences Centre Manchester United Kingdom; 7 School of Health and Wellbeing University of Glasgow Glasgow United Kingdom; 8 NHS Greater Glasgow and Clyde Glasgow United Kingdom; 9 Research Department of Clinical, Educational and Health Psychology University College London London United Kingdom; 10 North East London NHS Foundation Trust London United Kingdom; 11 The AVATAR2 PPI Group London United Kingdom

**Keywords:** auditory hallucinations, AVATAR therapy, peer research, psychosis, qualitative study

## Abstract

**Background:**

AVATAR therapy is a novel psychological therapy that aims to reduce distress associated with hearing voices. The approach involves a series of therapist-facilitated dialogues between a voice-hearer and a digital embodiment of their main distressing voice (the avatar), which aim to increase coping and self-empowerment.

**Objective:**

This study explored therapeutic processes that are distinctive to AVATAR therapy, including direct early work with voice content and the role of the therapist in dialogue enactment.

**Methods:**

People with lived experience relating to psychosis (peer researchers) contributed to each stage of the study. Peer researchers led semistructured interviews, which were conducted with 19 participants who received AVATAR therapy as part of the AVATAR2 trial, including 3 participants who dropped out of therapy. Data were analyzed using interpretative phenomenological analysis (n=5) and template analysis (n=14).

**Results:**

Participants described the initial challenges of experiential work with distressing voice content; however, most reported a meaningful increase in power and control over the course of dialogues and improvements with voices in daily life. A strong therapeutic alliance was experienced by all participants, including those who chose to discontinue therapy, often mitigating the discomfort associated with initial challenges by enhancing their sense of safety. Several important themes relating to individual engagement were highlighted, such as the emotional intensity of the experience and the importance of participants’ determination and open-minded attitudes despite initial doubts. Those who decided not to continue with therapy described challenges with the realism of working dialogically with a digital representation of their distressing voice.

**Conclusions:**

This study has provided a deeper understanding of the experience of engaging in AVATAR therapy, in particular the challenges and opportunities of direct work with voice content. The importance of therapeutic alliance and establishing a sense of voice presence has been emphasized. Implications for the planned optimization and wider implementation of AVATAR therapy in routine care settings are discussed.

**Trial Registration:**

ISRCTN Registry ISRCTN55682735; https://www.isrctn.com/ISRCTN55682735

## Introduction

Voice-hearing is a diverse experience that can occur in both clinical and nonclinical populations. While some voices may be experienced as neutral or positive, others are distressing, hostile, and disruptive to daily life [1,2]. Recent developments in psychological interventions for distressing voices have foregrounded the relationship between the voice-hearer and voice as a key treatment target [3-5]. AVATAR therapy is one such approach, which aims to reduce the distress associated with hearing negative voices [6]. In AVATAR therapy, voice-hearers are supported to create the voice and image of a digital avatar to represent their main distressing voice. There is flexibility to customize facial and vocal features, including nonhuman forms (eg, devil), and the avatar is voiced in real time by the therapist using voice-transformation software. The person interacts with the avatar in a series of dialogues, supported by the therapist and interspersed with preparatory and reflective discussions (including role play), with the aim of taking back power and control from the voice [7,8]. Further details on therapeutic processes and targets are described in the main trial [9] and Ward et al [8]. Early work [7] identified 2 therapy phases. The focus of phase 1 is on exposure to the avatar voice enactment (including verbatim voice content) and encouraging the voice-hearer to respond assertively. In phase 2, the avatar concedes power to the voice-hearer, and the focus shifts to other therapy targets, including self-esteem.

Building on Leff et al [6] work, this approach was tested in a fully powered randomized controlled trial (RCT; AVATAR1), which found that AVATAR therapy was more effective than supportive counseling in reducing the frequency, distress, and omnipotence of voices at 12 weeks post therapy with a large between-group effect size of 0.8 for overall voice severity [7]. Following this, a multicenter RCT (AVATAR2 [9]) has recently tested a 6-session version of AVATAR therapy (AVATAR-Brief [AV-BRF], comprising the key elements of exposure, assertiveness, and self-esteem) alongside an extended (12-session) form of AVATAR therapy (AV-EXT). The AV-EXT protocol was designed to foreground the understanding of voices within the person’s broader life history [10] and include a wider range of therapeutic targets, including trauma. The AVATAR2 trial found that voice distress (primary outcome) and voice severity significantly improved in both AV-BRF and AV-EXT at the end of therapy, compared to treatment as usual alone, with improvements maintained at 28 weeks but no longer statistically significant. There was a significant and sustained reduction in voice frequency for AV-EXT, but not AV-BRF. While both therapy groups showed significant improvements across a range of other secondary outcomes, AV-EXT showed a wider range of positive effects in areas including increased empowerment, voice understanding, and well-being, and these tended to be stronger and longer lasting [9].

While AVATAR therapy shares common therapeutic aims with other cognitive and relational therapies, there are several distinctive aspects, notably direct exposure to feared stimuli and real-time dialogues with a digital embodiment of the voice enacted by the therapist [8,11]. This includes the experience of “voice presence,” defined as the degree to which the dialogue with the avatar is experienced “as if” talking to the voice [12,13]. These unique features raise important questions around the blending of digital technology and relational therapy, particularly regarding the experience of therapeutic alliance (TA) within this distinct digital context, which differs from app-based interventions discussed in existing literature [14-17]. The participant experience of AVATAR therapy was examined within a previous qualitative study conducted as part of the AVATAR1 trial [11]. The use of technology was generally well accepted by participants, and the collaborative process of designing the avatar and enacting the relationship with the voice was reported to be helpful in facilitating therapeutic dialogue. Participants described feeling supported by their therapist and were able to identify specific learned strategies for managing voices, such as standing up to the voice and choosing to disengage. Although Rus-Calafell et al [11] provided important support for the acceptability of AVATAR therapy, limitations included the recruitment of only 1 (5%) therapy dropout and the inclusion of patient and public involvement (PPI) into some (eg, developing topic guide, conducting interviews) but not all research processes. It was recommended that future qualitative work should aim to build understanding of the specific challenges of early direct work with derogatory verbatim voice content given that this represents a central aspect of the AVATAR therapy approach and a potential point of difference from other psychological approaches.

This study differed in that it used interpretative phenomenological analysis (IPA), a qualitative approach that aims to understand the emotions and personal meanings associated with experiences in the context of an individual’s lifeworld [18,19]. IPA was selected to provide in-depth, idiographic insights into participants’ subjective experiences [20]. Template analysis is a form of thematic analysis that involves developing and refining a hierarchical coding template, often beginning with a priori codes, which is iteratively applied and modified across the dataset to organize themes in a meaningful way [21]. As Frost [22] highlights, interpreting data pluralistically using multiple methods of analysis can facilitate a more unbiased and holistic perspective of participants’ experiences. Template analysis can be incorporated alongside IPA to extend the scope of the analysis beyond an individual case level, enabling identification of convergences and divergences across participants, thus providing a more comprehensive understanding [23]. This dual approach of IPA and template analysis was used by Bond et al [24] to capture both the nuances of individual experiences and the broader shared themes, ensuring both depth and breadth [23]. Additionally, this analytic approach recognizes the potential influence of the researcher on interpretation [25], highlighting the value of a peer research approach to improve both the rigor and relevance of qualitative analysis [26]. Therefore, involving people with relevant lived experience, as demonstrated by Bond et al [24], can enrich the depth, sensitivity, and ecological validity of the analysis [27].

In this study, we aim to move beyond a validation of the acceptability of the approach to provide a rich investigation of therapeutic features that are distinctive to AVATAR therapy. Using peer methods across all stages of the study and improving representation of those who dropped out of therapy, we aim to address the following research questions:

- How did participants experience working directly with verbatim voice content?
- How was TA experienced by participants in the context of AVATAR therapy?
- What were the experiences of participants who decided not to continue with therapy?

## Methods

### Study Design and Setting

This qualitative study investigated participant experiences of AVATAR therapy based on semistructured interviews. Adopting a peer research approach, this study was nested within the AVATAR2 trial (ISRCTN55682735, registered on January 22, 2020), a multicenter RCT conducted across 4 trial centers in England and Scotland (Institute of Psychiatry, Psychology, and Neuroscience, King’s College London; University College London [UCL]; The University of Manchester; and University of Glasgow). The additional research ethics approval for this study was granted by the London-Camberwell St Giles Research Ethics Committee in December 2022 (reference 20/LO/0657).

In line with open science processes, a preregistration form (based on Haven et al [28] and Staniszewska et al [29]) detailing the study protocol was published on the Open Science Framework to ensure transparency and reliability [30].

### Patient and Public Involvement

A total of 16 PPI representatives (termed peer researchers in this study) were involved across all stages of this study, with representation from all 4 trial sites. Most individuals had lived experience of hearing distressing voices, with some having received AVATAR therapy as part of the AVATAR1 or AVATAR2 trial, as well as a small number of people who had cared for a loved one who had experienced this. This included (1) coproduction of participant-facing documentation, (2) collaborative development of the topic guide, (3) codelivery of interviews, and (4) contributions to data analysis. This peer research approach aligns with the National Institute for Health Research INVOLVE guidelines [31], which highlight 6 key values—respect, support, transparency, responsiveness, fairness of opportunity, and accountability. For example, all peer researchers were paired with a site research worker who provided flexible and personalized support in order to develop a collaborative working relationship [32]. Peer researchers were remunerated according to INVOLVE guidelines [31]. More details are provided in Multimedia Appendix 1, and PPI across the trial is further discussed in Owrid et al [32].

### Participants

We recruited participants who had received AVATAR therapy as part of the AVATAR2 trial [33]. This included those randomized to the AV-BRF and AV-EXT arms. All participants met eligibility criteria for the trial. Inclusion criteria were (1) aged ≥18 years, (2) under the care of a mental health team, (3) hearing a distressing voice in English for at least 6 months, (4) adequate English to take part, and (5) schizophrenia spectrum disorder or an affective disorder with psychotic symptoms. Exclusion criteria were (1) lacking capacity to consent, (2) currently undertaking individual psychological therapy for voices, and (3) currently experiencing an acute mental health crisis. In addition, specific inclusion criteria for this study were as follows: (1) received AVATAR therapy in the AVATAR2 trial, (2) able and willing to provide informed consent to take part in the interview, and (3) willing to have the interview audio-recorded.

### Sampling and Recruitment

As IPA principles require homogeneity around a phenomenon of interest [19,34], this was implemented in terms of adults experiencing distressing voices who have received AVATAR therapy. A predetermined recruitment strategy was consistently followed by trial coordinators at each site. This involved initially using consecutive sampling methods to invite participants for interview who had recently completed the final phase of the AVATAR2 trial. To achieve representativeness across sample characteristics and therapy delivery, purposive sampling methods were then applied to ensure that (1) the sample represented all 4 trial sites, (2) the gender and ethnicity of the sample were representative of participants across the trial, (3) the sample represented both AV-BRF and AV-EXT therapy arms, (4) the interviews covered experiences of working with a range of therapists, and (5) those recruited included a proportionate number of those who did not complete the intervention.

The aim was to recruit 20 participants across the 4 trial sites, including 4 participants who dropped out of therapy.

### Interview Guide

Semistructured interviews were guided by a topic guide (Multimedia Appendix 2) that was developed iteratively in collaboration with peer researchers, trial coordinators, therapy leads, and site research workers. Key areas of questioning were based on the research questions and initially drafted by the study team in line with IPA principles, such as focusing on the individual’s emotional experiences and associated meanings. The guide was then revised across 4 consultations, involving a total of 5 peer researchers. For example, questions were reworded to ask about “the voices” instead of “your voices” to ensure destigmatizing and nonjudgmental language. Interview questions explored participants’ (1) experience of dialoguing with a representation of their distressing voice, (2) TA formed with the therapist, and, where applicable, (3) reasons for not completing therapy.

### Interview Process

Interviews were conducted either in person, online via Microsoft Teams, or via telephone, depending on participant preference. All interviews were conducted by a site research worker and peer researcher. In line with Harding et al [35], peer researchers took the lead in interviewing all participants while the accompanying site research worker provided support and input as required. Peer researchers disclosed their relevant lived experience when introducing themselves, but it was at their discretion how much detail they shared during the interview. A total of 14 peer researchers were involved in leading interviews, all of whom attended training (Multimedia Appendix 3), role-play practice, and supervision. The lead author, ERE, was available to peer researchers, site research workers, and trial coordinators for support with all aspects of protocol delivery to ensure a standardized approach.

### Analysis

Data were analyzed using a dual approach of IPA and template analysis, as described by Bond et al [24,36]. Nineteen is a relatively large sample for IPA’s rigorous and idiographic approach. Therefore, a small core sample of 5 (26.3%) transcripts was included in IPA processes, with analysis then extended across the remaining transcripts using template analysis. In line with IPA principles, the selection of transcripts was based on the richness of data and ensured diversity in trial sites, gender, and ethnicity. To ensure homogeneity, as required for IPA [19,34], all selected transcripts were from therapy completers. This selection process was led by ERE with guidance from experts in qualitative research methods and input from peer researchers who conducted interviews.

IPA followed analytic processes described by Smith et al [18] and Smith and Nizza [37]. The 4 peer researchers who conducted the 5 selected interviews first listened to their interview recordings to reflect on their emotional responses and perspectives of participants’ experiences. ERE then met individually with each peer researcher in 1-hour consultations to discuss interpretations. Using a case-by-case approach, line-by-line annotations were made for each transcript, incorporating the peer researcher’s perspectives and noting descriptive, linguistic, and conceptual comments. Identified meanings were therefore informed by peer researchers’ interpretations and then formulated into experiential statements, which were subsequently clustered and interpreted for each case. Convergences and divergences of themes were then considered across cases, generating group experiential themes and subthemes. Excerpts of the analytic process are presented in Multimedia Appendix 4.

Before applying template analysis, the resulting coding structure (ie, group experiential themes and subthemes) alongside illustrative quotes was reviewed by 6 peer researchers across 4 consultations to develop the provisional template (Multimedia Appendix 5). This input extended initial interpretations by drawing on lived experience, for example, emphasizing the complexity of the avatar-voice association, highlighting the profound nature of participants’ emotional experiences, and helping to identify language that felt authentic. This template was then used to guide template analysis of the remaining transcripts, following the processes outlined by King et al [21]. Transcripts were introduced in subgroups, focusing first on therapy completers and, second, on those who dropped out of therapy. This clearly identified where these 2 subgroups aligned and where new experiences arose. Where data diverged, new codes were assigned, and the template was changed to accommodate these perspectives. Once all transcripts had been coded and the template had been developed iteratively, all transcripts were reviewed again to ensure reliability.

Furthermore, 4 peer researchers reviewed the finalized template and contributed to refining codes, wording themes, and interpreting results. For example, the subtheme initially named “Difficulties generalizing learning” was reworded to “Difficulties translating changes to voices” to ensure accessible, nonclinical language. Additionally, the subthemes “Initial doubts and anxieties” and “Positive, open-minded attitudes” were merged into “Open-minded attitudes despite initial doubts” to reflect peer researchers’ interpretation that open-mindedness acted as an overriding force against early anxieties.

IPA and template analyses were conducted by ERE with input from peer researchers across the analytic process, ensuring that lived experience actively contributed to meaning-making. The outcome of this dual approach was a structure of key themes and subthemes (ie, the finalized template) representing all 19 interviews, including the 5 transcripts analyzed using IPA.

### Reflexivity

All members of the AVATAR2 team reflected on the perspectives they brought to the study design, conduct, and analysis through whole team meetings, peer group supervision with site research workers and peer researchers, and the lead author’s reflective research journal. The team approach and wider context of the qualitative research program were published on the Open Science Framework [30]. The lead author (ERE), a White British female trainee clinical psychologist, had no involvement in the AVATAR1 trial nor in the wider AVATAR2 trial, as her role was limited to this qualitative study, and had no prior contact with participants. While her clinical experience of working with people with psychosis, including delivering cognitive behavioral therapy, may have fostered positive attitudes toward psychological therapy, she had no clinical experience specifically with AVATAR therapy. Completing the Jacobson and Mustafa [38] positionality map supported reflection on social identity and potential sources of bias.

### Ethical Considerations

This study was approved by the London-Camberwell St Giles Research Ethics Committee in December 2022 (reference 20/LO/0657) and was conducted in accordance with the principles of the Declaration of Helsinki.

The participants in this qualitative study signed informed consent before participating in the interview that included consent for publication. All potentially identifiable information has been removed from published material included in this study. All interviews were audio-recorded, transcribed, and anonymized. Participants were paid £20 (US $26.65) for taking part in the interview.

## Results

### Participants

A total of 19 participants took part in qualitative interviews, which were conducted between July and September 2023 and took place between 11.4 and 96.4 (mean 53, SD 29.1) weeks after their final AVATAR therapy session. Overall, 6 (31.6%) participants were recruited from King’s College London, 4 (21.1%) from UCL, 4 (21.1%) from Manchester, and 5 (26.3%) from Glasgow. Demographic and treatment information of participants are presented in Table 1 alongside that for the whole study sample of those who received AVATAR therapy (AV-BRF and AV-EXT). Participants in the interview sample were broadly representative of the wider sample; however, differences were not statistically tested.

**Table 1:** Demographic and treatment information.

Variable		Whole sample (n=230)	Interview sample (n=19)
Age (years), mean (SD; range)		40.07 (13.49; 18-70)	38.63 (14.06; 19-66)
**Gender, n (%)**			
	Men	143 (62.2)	13 (68.4)
	Women	85 (37)	6 (31.6)
	Other	2 (0.9)	0 (0)
**Ethnicity, n (%)**			
	White	136 (59.1)	11 (57.9)
	Black Caribbean	13 (5.7)	0 (0)
	Black African	21 (9.1)	2 (10.5)
	Black Other	7 (3)	0 (0)
	Indian	5 (2.2)	1 (5.3)
	Pakistani	8 (3.5)	0 (0)
	Chinese	2 (0.9)	1 (5.3)
	Other	38 (16.5)	4 (21.1)
**Treatment arm, n (%)**			
	Brief	116 (50.4)	10 (52.6)
	Extended	114 (49.6)	9 (47.4)
**Dropout, n (%)**			
	Yes	69 (30)	3 (15.8)
	No	161 (70)	16 (84.2)

A total of 9.9% (16/161) of therapy completers and 4.3% (3/69) of those who discontinued therapy are represented in the sample. Of those who dropped out of therapy, 2 participants decided not to continue after 1 session prior to any avatar dialogue, and 1 participant dropped out after 8 sessions (including 5 avatar dialogues). The 3 participants who dropped out, Grace, James, and Alexander (pseudonyms), were all allocated to AV-EXT.

### Key Findings

Integrating IPA and template analysis processes, 4 overarching themes were identified, from which a total of 14 subthemes emerged. Details of themes alongside the number of participants are represented in Table 2 and subsequently presented in more depth. This is summarized in Multimedia Appendix 6. Illustrative quotes are presented within the text and in Multimedia Appendix 7. Pseudonyms are used throughout.

**Table 2:** Themes and subthemes identified, with participant counts.

Themes and subthemes			Sample, n (%)		Completers, n (%)		Noncompleters, n (%)
**Shift in relationship with avatar and, consequently, voices**							
	Initial challenges adjusting to avatar	18 (94.7)		15 (93.8)		3 (100)	
	Collaborative efforts facilitated meaningful connection to avatar	14 (73.7)		13 (81.3)		1 (33.3)	
	With therapist support, participants felt empowered to stand up to avatar	16 (84.2)		15 (93.8)		1 (33.3)	
	Positive shift with voices	15 (78.9)		13 (81.3)		2 (66.7)	
**Crucial role of person-centered therapist**							
	Felt safe, supported, and understood	19 (100)		16 (100)		3 (100)	
	Person-centered flexibility	15 (78.9)		13 (81.3)		2 (66.7)	
	Significant impact of therapeutic alliance	16 (84.2)		14 (87.5)		2 (66.7)	
**Individual approach and experience**							
	Open-minded attitudes despite initial doubts	18 (94.7)		15 (93.8)		3 (100)	
	Determination facilitated engagement and outcomes	17 (89.5)		16 (100)		1 (33.3)	
	Profound emotional experience	17 (89.5)		14 (87.5)		3 (100)	
	Offered novel approach to tackle voices	12 (63.2)		11 (68.8)		1 (33.3)	
**Barriers to engagement and outcomes**							
	Emotional challenges with avatar	10 (52.6)		7 (43.8)		3 (100)	
	Not the right approach for the individual at that time	8 (42.1)		5 (31.3)		3 (100)	
	Difficulties translating changes to voices	6 (31.6)		6 (37.5)		0 (0)	

### Theme 1: Shift in Relationship With Avatar and, Consequently, Voices

This theme explores how participants’ experiences of working dialogically with verbatim voice content evolved across therapy. The subthemes “Initial challenges adjusting to avatar” and “Collaborative efforts facilitated meaningful connection to avatar” focus on initial adjustment processes. Subsequently, the subtheme “With therapist support, participants felt empowered to stand up to avatar” explores communication in dialogues, and the final subtheme, “Positive shift with voices,” highlights improvements in coping.

#### Initial Challenges Adjusting to Avatar

Being exposed to derogatory voice content through the avatar could be challenging at first, with initial reactions ranging from fear to emotional disconnection. For 5 participants, the avatar immediately provided an accurate representation and felt real, evoking a similar emotional response to hearing voices. This could lead to heightened anxiety.

That was probably one of the most difficult days of my life, that very first session [...] I was a just a complete utter mess that day.

Charlotte, AV-BRF, completed therapy

Conversely, 4 participants experienced emotional disconnection due to discrepancies between the avatar and the voices. Accurately matching these elements was a common challenge, and many felt it was initially difficult to fully capture both the content and intensity of such a dynamic and individualized experience.

It was off-putting [...] it was harder for me to really engage with it.

Stephen, AV-BRF, completed therapy

This was a spectrum of experience, and most reported only short-term discomfort within early sessions. In particular, 6 participants described that it was initially strange to experience the familiarities of verbatim voice content in a new context.

Rather than just, you know, in my mind, it was actually there in front of me.

Grace, AV-EXT, dropped out of therapy

This tangible representation appeared particularly confronting for participants who had used avoidance to cope.

I was pretty nervous to make things that I didn’t want to be real seem more real.

Gabriel, AV-EXT, completed therapy

Fears arose that the avatar might merge with other distressing experiences. In fact, this did occur for Gabriel, although he highlighted that this was ultimately useful for his engagement and overcoming difficulties, as it connected his experience of the avatar to his experience of voices.

The face and the voice of the avatar then becoming sort of combined with the other things that I was seeing and hearing at the time.

Gabriel, AV-EXT, completed therapy

Therefore, initial exposure to the avatar was a complex and dynamic experience. For instance, Stephen initially struggled with the software, particularly as he felt customization options for creating a Black female face and voice were limited, so felt emotionally detached from the avatar as it did not accurately represent his voice experience. However, following the first session, he noticed increased voices in daily life. While temporarily challenging, this helped him make a stronger link between the avatar and his voice experience and subsequently enhanced his emotional engagement with the avatar.

In the time of doing it, I didn’t really feel anything from it, it felt very gimmicky [...] as soon as I’d finished the session then the voices kind of hit me and they were quite intense.

Stephen, AV-BRF, completed therapy

For 5 participants, these challenges with heightened anxiety and emotional disconnection impacted early dialogues, leading to feeling unable to respond to the avatar or subconsciously reverting to ignoring voice content.

Feelings of almost of being choked out, I couldn’t really think straight.

Ishan, AV-EXT, completed therapy

Went into default mode of just ignoring it.

Stephen, AV-BRF, completed therapy

#### Collaborative Efforts Facilitated Meaningful Connection to Avatar

Adjusting and collaboratively working through initial challenges was crucial to relate to the avatar as if they were relating to the voice. Nine participants reported that increased exposure over time improved familiarity and comfort levels.

As I did one or two sessions, I just got used to it and [...] I knew what was going to happen.

Zahid, AV-BRF, completed therapy

Participants learned to accept any inaccuracies and instead focus on the experience of interacting with the avatar. Three participants described making efforts to overlook discrepancies and using their imagination to “fill in a lot of blanks” in order to relate the avatar to the voice.

I realised it really wasn’t about being dead accurate to what the voices was sounding like but more about the interaction with it.

Ishan, AV-EXT, completed therapy

Therapists played a crucial role in adjusting software based on participant feedback and enacting the avatar to represent the voice both authentically and respectfully.

My therapist was so willing and helpful with that and presented that in a way that was respectful but also true to my experience, made it very visceral and real for me.

Stephen, AV-BRF, completed therapy

As verbatim voice content was often derogatory, this involved open and ongoing conversations to ensure informed consent. Individualized discussions around therapy aims and processes helped participants to understand and engage meaningfully. For Joshua, creating a story for the avatar enhanced his engagement. As the avatar represented the voice of God, his therapist supported him to frame the voice’s aggression as stemming from a lack of understanding about human experience. Therefore, dialogues were used as a space for him to explain to the avatar the complexities of being human, with the aim of helping the avatar to understand him better and reduce its anger.

We started to kind of like develop [...] a narrative to the avatar and like understand the avatar in a way.

Joshua, AV-BRF, completed therapy

Collaborative efforts improved most participants’ connection to the avatar, facilitating engagement and meaningful dialogues.

Once you get that, it’s so real and it has such a profound effect that it doesn’t necessarily matter whether it matches the voice.

Stephen, AV-BRF, completed therapy

However, challenges did persist for the 3 therapy completers who did not perceive notable benefits from therapy (Zahid, Georgia, and Mai Su), and they continued to struggle to connect the avatar to the voice.

Getting the two together was [...] the difficult bit.

Zahid, AV-BRF, completed therapy

#### With Therapist Support, Participants Felt Empowered to Stand Up to Avatar

Most participants experienced a gradual but distinct shift in power dynamics across therapy. Initially, many felt overpowered by the avatar, mirroring their day-to-day struggles with voices, but 14 participants described gaining confidence over time. This was frequently conceptualized as a “battle” in which participants fought back and progressively gained control.

It actually felt quite good to stand up to it [...] I’ve just let him use me as a big punch bag, but with AVATAR therapy, I felt as if I was gaining more control.

Charlotte, AV-BRF, completed therapy

For 4 participants, focusing on the avatar’s face helped them feel more assertive, as they could direct their communication to something specific and externalized. The process of responding to the avatar was highly individualized, depending on the context and relationship with the voice. For some, it involved learning to disengage, while others focused on more compassionate communication.

It really wasn’t about winning; it was sort of about leaving the conversation with the avatar at an agreeable sort of level.

Ishan, AV-EXT, completed therapy

Therapist support before, during, and after dialogues was identified as crucial in facilitating positive change. This included role-play, reassurance, and reflective discussions. The therapist’s unique position as both a participant and observer within dialogues was distinctly valued in facilitating new insights.

I think the confidence grew basically just by practising and [...] talking after the actual avatar session with the therapist.

Ishan, AV-EXT, completed therapy

#### Positive Shift With Voices

As participants gained confidence in confronting the avatar, many observed mirroring of improvements with voices. The metaphor of a battlefield arose again as participants compared being “in the trenches” prior to therapy to now equipped to fight back, such as with “a shield to [*...*] block out the voices.”

I’m able to answer them back, in a much more direct way that I used to.

William, AV-EXT, completed therapy

A total of 13 participants reported reductions in voice frequency and severity due to improved confidence and abilities to cope with voices. Consequently, 9 described improved quality of life, such as increased social interaction made possible by the reduced impact of voices. In addition, 8 participants reported an improved understanding of voices supported by discussions with the therapist, which helped them to accept and reframe their experiences and develop effective coping strategies. Although voices persisted for all participants, their impact notably reduced for most.

They’re not as bad now, they kind of sit back and leave me alone.

Caleb, AV-EXT, completed therapy

Skills and insights gained could have a lasting impact, and 6 participants highlighted the importance of continued practice to maintain positive changes. Experiences in AVATAR therapy often had a ripple effect across participants’ lives, fostering inner strengths and rebuilding self-esteem.

I felt empowered that like if I could get through that, then there’s not really much that I can’t get through.

Stephen, AV-BRF, completed therapy

### Theme 2: Crucial Role of Person-Centered Therapist

The role of the therapist in AVATAR therapy was emphasized. The subthemes “Felt safe, supported, and understood” and “Person-centered flexibility” highlight valued aspects of the therapist’s approach and qualities, and the subtheme “Significant impact of TA” explores the effect of the therapeutic relationship on engagement and outcomes.

#### Felt Safe, Supported, and Understood

Initial discomfort, particularly when sharing verbatim voice content, was prevalent due to fears of stigma, misunderstanding, and judgment, often compounded by negative past experiences.

I find it really difficult to talk about him and that’s why I try and keep him in, because I don’t want other people judging.

Charlotte, AV-BRF, completed therapy

Despite these concerns, all participants described feeling secure and supported early in therapy, which facilitated opening up. Professional boundaries, confidentiality, and therapist credentials contributed to this sense of safety.

I felt like it was a safe environment, it was quite enclosed and isolated from other people.

Ishan, AV-EXT, completed therapy

A total of 18 participants highlighted the importance of core therapeutic skills, such as empathy, transparency, and clear communication. Participants felt heard and accepted without judgment, fostering a trusting therapeutic relationship and providing a valued contrast to past experiences.

I could feel the empathy and she wasn’t sympathetic, but her empathy was there and [...] I was able to confide in her.

Paula, AV-BRF, completed therapy

In addition, 13 participants also described feeling deeply understood, enhanced by therapists’ specialist knowledge about voice-hearing. In fact, it was identified how therapists developed a distinct depth of understanding through direct exposure to voice content via the avatar. Gaining an ally in this way could feel validating.

I feel like there’s only so much somebody can really understand until they’re literally being face-to-face [...] to role play that with me, I feel that there’s a certain level of understanding that you get from that that you wouldn’t get from other interventions.

Stephen, AV-BRF, completed therapy

#### Person-Centered Flexibility

A total of 14 participants highlighted that flexibility and collaboration empowered them to engage, as they felt involved as an equal partner. Unpressured, person-centered pacing and choice were particularly valued, especially surrounding avatar dialogues.

Always make sure I had a say, if I was able to continue, if I wanted to stop, if I was able to do it, whatever [...] nothing was imposed on me.

Paula, AV-BRF, completed therapy

Central to this experience, therapists remained attuned to participants’ emotional and psychological states, so they were flexible in responding to individual needs.

Could tell when I was not having a good day, which was important to me [...] they recognised that and were able to work around it, which really helped me.

Matthew, AV-EXT, completed therapy

Participants also valued therapist flexibility in offering space to discuss issues outside of avatar dialogues, which helped to explore alternative interpretations of their experiences and develop new insights.

He was really, really helpful and flexible and just let me speak about whatever I wanted to speak about.

Gabriel, AV-EXT, completed therapy

Collaborative use of other relevant materials, such as psychoeducation, was highlighted by 4 participants for its value in reducing stigma and enhancing understanding.

We went through a document on different types of intrusive thoughts people have and I related to quite a lot of them [...] so it does make me feel a bit more normal.

Joshua, AV-BRF, completed therapy

Additionally, 8 participants valued therapists being flexible in rescheduling appointments, arranging transport, offering remote options, and making check-in calls between sessions.

There was a flexibility [...] that allowed me to be able to complete.

Stephen, AV-BRF, completed therapy

It felt important for therapists to embrace the participant’s individuality, understand their identity, and acknowledge unique needs, strengths, and values. This person-centered approach enhanced TA, engagement, and outcomes.

I was allowed to be myself even while trying to get help.

Paula, AV-BRF, completed therapy

People are so simple but yet so incredibly complex like the human mind is simple: [...] input, process, output. But our souls and what we do, how we live, everything inside it, that life and mind confuses things and complicates things because that’s where the differences come.

Alexander, AV-EXT, dropped out of therapy

#### Significant Impact of TA

TA was a central aspect of therapy, with 14 participants noting its importance for experience, engagement, and outcomes. Trusting the therapist was essential for participants to remain engaged despite the emotional challenges of working with verbatim voice content.

I was able to trust the process and trust her, but I had to trust her to trust the process.

Paula, AV-BRF, completed therapy

Strong TA could also mitigate distressing aspects of therapy, such as voices responding negatively to progress in dialogues.

The voices themselves [...] were like dismissive of it [...] but when you have someone that [...] understands you, it does make you feel better, and the voices don’t- can’t change that.

Joshua, AV-BRF, completed therapy

While not identified as a notable issue for most, 2 participants described difficult feelings in relation to knowing that it was the therapist, someone they trusted, voicing derogatory voice content and reenacting abuse within avatar dialogues, particularly given the level of personal importance attached to the therapeutic relationship. Additionally, 4 participants highlighted that TA facilitated a clear separation between the avatar and the therapist.

Sometimes, knowing it’s her that’s saying it [...] it hurt a bit [laughs] because I liked her.

Charlotte, AV-BRF, completed therapy

The experience of TA itself had a positive impact in the short- and long-term, enhancing confidence and self-compassion and reducing stigma and self-blame.

The experience was empowering for me because I felt [...] I was not a patient being given treatment, I feel like I was treated as an equal.

Stephen, AV-BRF, completed therapy

This positive interpersonal experience reduced loneliness and improved trust in other relationships.

Beginning to gain trust again, it was slow, but it definitely opened the door to trusting again.

Paula, AV-BRF, completed therapy

Even those who did not complete therapy found this relationship impactful, and the therapy completers who did not perceive notable benefits (Zahid, Georgia, and Mai Su) found value in speaking openly with their therapist despite feeling that the AVATAR approach was not right for them at the time, demonstrating the importance of TA. For example, Alexander had previously lost hope that things could get better for him, as he felt no one cared, so this experience presented a significant catalyst for change.

People being genuinely supportive and actually trying to help people like me was enough for me. It felt like, no, maybe people do actually care, which was one of the reasons why I gave up in the first place.

Alexander, AV-EXT, dropped out of therapy

### Theme 3: Individual Approach and Experience

This theme emphasizes the impact of individual participants’ approaches and experiences. The subthemes “Open-minded attitudes despite initial doubts” and “Determination facilitated engagement and outcomes” highlight personal strengths. The subtheme “Profound emotional experience” focuses on the depth of emotional experience. The subtheme “Offered novel approach to tackle voices” considers the novelty of the therapy approach to the individual.

#### Open-Minded Attitudes Despite Initial Doubts

Most participants entered AVATAR therapy with some degree of anxiety, ranging from mild worry to skepticism.

Nervous [...] a bit scared.

Georgia, AV-BRF, completed therapy

Concerns included doubts about AVATAR therapy’s effectiveness, the computerized approach, and fears that working directly with verbatim voice content might trigger negative psychological experiences.

I was very hesitant and reluctant to do it because, due to the nature of my voices, I had spent an extensive amount of time ignoring them and not engaging with them [...] so there was a fear of being like triggered or traumatised.

Stephen, AV-BRF, completed therapy

A total of 17 participants highlighted open-mindedness as central to initial engagement. Attitudes varied, and hope was often held cautiously; however, most were motivated to “give it a go,” often driven by past challenges in accessing psychological support.

I just thought it doesn’t hurt to give it a try.

Zahid, AV-BRF, completed therapy

Although 10 participants emphasized the central role of internal motivations, support from friends and family and trust in referring clinicians could enhance willingness to engage. Conversely, James described feeling pushed by his mental health team to participate despite his own reservations, which was disempowering and negatively impacted his engagement.

I wasn’t really given a choice. I was told that I would want to do it, so therefore I should. And so I was just kind of shunted onto it.

James, AV-EXT, dropped out of therapy

#### Determination Facilitated Engagement and Outcomes

Determination was identified as important for maintaining engagement and facilitating positive outcomes for 17 participants. Motivations typically centered on a drive to overcome distressing voices and make progress, even when therapy processes felt uncomfortable.

It was just because I had a strong motivation to get past it all.

Asim, AV-BRF, completed therapy

Noticing gradual improvements could feel meaningful and reinforce motivations to continue. Paradoxically, Stephen found that noticing symptoms initially worsen actually increased his resolve to persevere.

The intensity of that after the first session was [...] trying to dissuade me, but I’d already made my mind up that I was going to do this and the fact that it did happen [...] gave me more motivation to continue to lean into it more.

Stephen, AV-BRF, completed therapy

Determination went beyond physically attending sessions, as 9 participants emphasized proactive efforts to be vulnerable and fully participate. In this way, the therapy experience and outcomes were perceived to be contingent on the depth of engagement and commitment to processes involved.

I think it varies what you put into it [...] that’s what makes a big difference.

William, AV-EXT, completed therapy

However, engagement could be negatively impacted by external life stressors, which disrupted regular session attendance and motivations to keep going.

I’m glad I didn’t have to do the 12-week sessions because that would have been quite hard just to fit in with all my [...] problems I had.

Mai Su, AV-BRF, completed therapy

#### Profound Emotional Experience

AVATAR therapy elicited a range of emotional experiences, which could be complex and difficult to articulate. Therapy was described as a meaningful journey by 15 participants, and the experience often felt transformative in that completing therapy led to personal growth, insights, and even a “paradigm shift.”

It felt like, you know when people go on TV shows and they do social experiments, and then it’s like this has changed my life like exponentially? It felt like I’ve been through this weird experience that I wouldn’t change for the world.

Stephen, AV-BRF, completed therapy

Relevant to this meaningful journey, the emotional demands of therapy also presented challenges for 13 participants, such as reliving traumatic memories, confronting underlying problems, and feeling overwhelmed.

Difficult feelings of reliving what had happened to me [...] what I had put to the back of my mind was all coming out.

Grace, AV-EXT, dropped out of therapy

A total of 8 participants felt the depth of emotional experience enhanced outcomes, perceived as a challenging but necessary aspect of directly working through core issues. In this way, while experiences often became initially and temporarily more difficult, this eased across therapy and contributed to a meaningful sense of achievement when emerging stronger.

I really did feel like I was going back to the worst place [...] that I’ve been in terms of voices [...] that was a bit difficult but it passed [...] and I’m glad that it happened because I know how to deal with that better now.

Gabriel, AV-EXT, completed therapy

Additionally, therapy ending brought up mixed feelings. Some participants described a sense of closure and readiness to continue their recovery independently, while others experienced sadness, loss, and difficulties adjusting.

I felt complete.

Stephen, AV-BRF, completed therapy

I was sad, goodbyes are always hard for me.

Caleb, AV-EXT, completed therapy

#### Offered Novel Approach to Tackle Voices

The unique approach of working directly with voice content within avatar dialogues was highlighted as central to the success of therapy by 7 participants. Many valued the opportunity to experiment with different tactics, learn new skills, and practice responding to voices in real time, which was often a novel experience.

It gave me new perspectives on how to approach managing voices, like different ways to test out what would work for me.

Gabriel, AV-EXT, completed therapy

Some had never considered directly responding to voices, while others found avatar dialogues provided the opportunity to experience voices differently and achieve more effective communication. The avatar diverging from voices and conceding power could open new conversations and lead to voices also shifting. In this way, 11 participants valued the novel approach AVATAR therapy offered.

I would never have been able to have had this type of level of connection with the voice because my personal experience of it would never give that response.

Stephen, AV-BRF, completed therapy

However, Zahid felt AVATAR therapy offered him nothing significantly new, and although the avatar conceded power, this did not have any significant impact on voices.

It wasn’t anything different that I haven’t done.

Zahid, AV-BRF, completed therapy

### Theme 4: Barriers to Engagement and Outcomes

The subthemes “Emotional challenges with avatar” and “Not the right approach for the individual at that time” focus on barriers and challenges faced by participants who dropped out of therapy, also relevant to some therapy completers. The subtheme “Difficulties translating changes to voices” relates to participants who completed therapy but struggled to access positive outcomes.

#### Emotional Challenges With Avatar

While most participants described early difficulties that eased over time (as discussed in “Initial challenges adjusting to avatar”), for some these were experienced as more significant emotional challenges. Such difficulties with digital representation could be challenging for some therapy completers to navigate, even among those who benefited, leading them to initially question their ability to continue.

Initially I found the avatar very difficult [...] I questioned whether I actually wanted to go through with it.

Matthew, AV-EXT, completed therapy

For the 3 completers who did not perceive benefit (Zahid, Georgia, and Mai Su), the avatar continued to feel either disconnected or frightening.

The avatar got a bit soft and started [...] back down a bit so it wasn’t quite true to life, so I don’t know.

Mai Su, AV-BRF, completed therapy

It was very deep.

Georgia, AV-BRF, completed therapy

All 3 participants who discontinued therapy (Alexander, James, and Grace) encountered emotional difficulties when working with a digital representation. Alexander reported feeling emotionally disconnected while cocreating the avatar, so he chose to drop out of therapy prior to any dialogues.

They are not the same. I was talking to a stranger. [...] There was no connection there at all.

Alexander, AV-EXT, dropped out of therapy

Although James also did not experience any avatar dialogues before deciding to discontinue therapy, he described the “crude” representation as an impassable barrier while acknowledging that the concept behind AVATAR therapy may be useful for some.

Freaked me out talking to this computer-generated head on the screen. It didn’t look like anything that I [pause] knew and so I just [...] left because I couldn’t engage in therapy with an avatar.

James, AV-EXT, dropped out of therapy

Grace found the avatar distressing and struggled with the emotional intensity. While she found the avatar a realistic representation and was able to meaningfully access therapy processes, she was fatigued by the sustained emotional intensity of dialogues and associated memories. This led her to drop out after 8 sessions, which involved 5 avatar dialogues.

I just felt I couldn’t talk any longer because it was just too deep and I just I was getting home at night and I was just crying and I thought I just can’t cope with this.

Grace, AV-EXT, dropped out of therapy

#### Not the Right Approach for the Individual at That Time

It was acknowledged that AVATAR therapy may not be suitable for everyone, depending on mental state and stage in recovery. For Alexander, choosing to discontinue therapy was experienced as empowering. The act of seeking help and developing a trusting relationship with his therapist provided a catalyst for change, emphasizing his inner strengths and jumpstarting his own path toward recovery. At this point, he felt therapy would be an unhelpful distraction, particularly in the context of autism and challenges with social interaction.

I was looking for the help and, as it turns out, the help wasn’t outside, it was inside.

Alexander, AV-EXT, dropped out of therapy

Grace wondered if AVATAR therapy may have been easier for her to navigate earlier in her recovery and expressed a desire to learn strategies for managing difficult emotions, although she gained meaningful benefits from the 8 sessions (5 dialogues) she attended.

I don’t think they could have done anything else, it was just up to me, I had to see what was good for me, what was not good for me.

Grace, AV-EXT, dropped out of therapy

Some therapy completers (n=5) suggested that effectiveness may vary depending on factors such as duration of voice-hearing experiences and readiness for therapy demands. In the context of their own challenges in early dialogues, it was speculated that working directly with voice content may be particularly difficult for those with “severe voices” and those who cope using avoidance.

I think it might just be a bit too overwhelming depending on the stage of and the seriousness of the voices.

Ishan, AV-EXT, completed therapy

Therefore, the importance of informed choice was emphasized, particularly given James’ experiences of feeling pressured by his referrer. Some mentioned that they were given insufficient information about what to expect, and while acknowledging it may be difficult to grasp what it will be like until directly experiencing it, they suggested that showing a brief video of an avatar dialogue could help to reduce uncertainty.

Although everything’s been explained, the words didn’t help. I had to actually do it to find out.

Alexander, AV-EXT, dropped out of therapy

#### Difficulties Translating Changes to Voices

A barrier experienced by the 3 therapy completers who did not report notable benefits related to issues translating improvements from avatar dialogues to day-to-day interactions with voices. Zahid, Georgia, and Mai Su were able to stand up to the avatar but did not experience improvements with voices. Mai Su described struggling with independent practice and reverting to default responses.

When I’m at home and the voice talks to me and it’s loud and I just don’t remember any of those sessions, so I just scream back it.

Mai Su, AV-BRF, completed therapy

A total of 3 therapy completers who reported benefit from AVATAR therapy also faced challenges in independently applying the approach to voices, experiencing initial discomfort and hesitancy.

I didn’t really practice too much [...] because I needed to build up more confidence [...] I felt like I wasn’t ready yet in myself.

Ishan, AV-EXT, completed therapy

Progress was not always smooth or cumulative. For example, Asim found voices increased in aggression after completing therapy and valued an additional session with his therapist to regain control. This highlights that, even when AVATAR therapy is beneficial, there may be challenges in applying learning to daily life, and there could be benefits of booster sessions.

After I started using the AVATAR therapy, you know, the stuff I learned, it was okay for a while but it came back angrier.

Asim, AV-BRF, completed therapy

**Figure 1:**
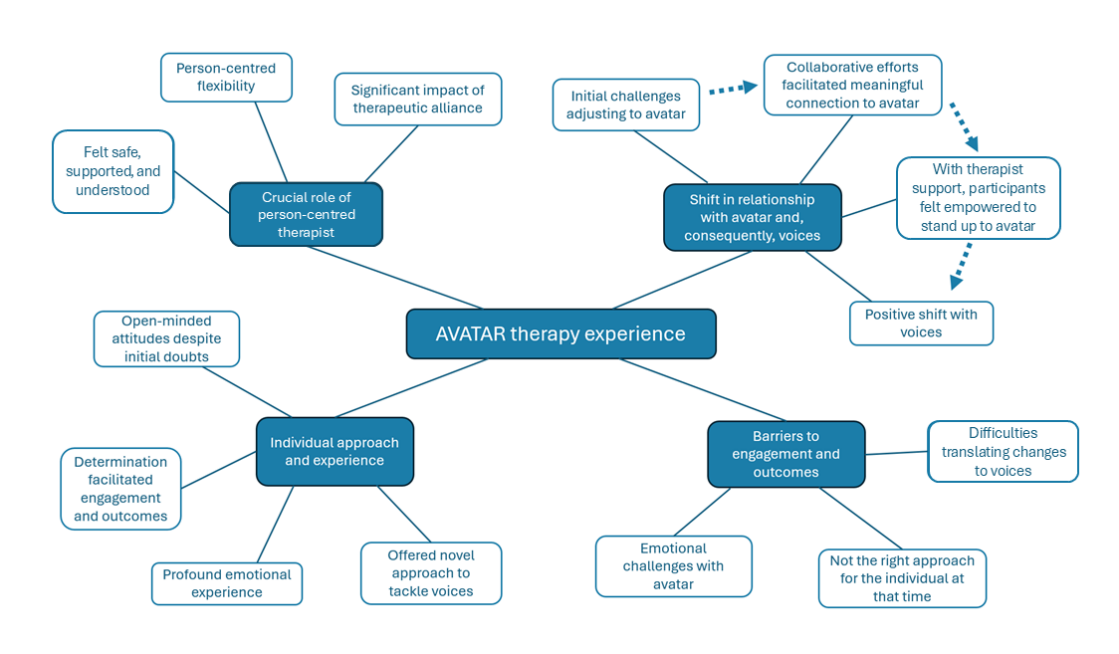
Thematic network.

The network of themes and subthemes is summarized in Figure 1, which illustrates the 4 overarching themes and their interrelationships. The arrows between subthemes in the “Shift in relationship with avatar and, consequently, voices” theme highlight the sequential process of first adjusting to and building a connection with the avatar before standing up to it and gaining benefits.

## Discussion

### Overview

This study used peer research methods and a dual approach of IPA and template analysis to explore the experience of AVATAR therapy, with a focus on direct work with voice content and the role of TA. Findings align with many of the themes identified in AVATAR1 qualitative work [11], particularly in highlighting the value of communicating with a digital representation, learning strategies, and the role of therapist support. Those who discontinued therapy described challenges with the realism of working dialogically with an avatar, reflecting that this approach may not be effective for everyone.

### How Did Participants Experience Directly Working With Verbatim Voice Content?

Despite initial challenges with early exposure and in vivo communication, working dialogically with voice content led most to experience an increased sense of power and control over voices, consistent with other cognitive approaches for distressing voices [39]. Participant accounts aligned with core therapeutic aims and processes, particularly around empowerment and self-esteem [8,40]. Consistent with inhibitory learning theory [41], participants described challenging distressing beliefs and targeting safety behaviors (including submission) within avatar dialogues, resulting in reduced anxiety over time. Positive outcomes were varied and individualized, including reduced voice frequency and omnipotence, enhanced understanding and acceptance, and improved interpersonal functioning. This aligns with relational approaches for distressing voices [3,7,42] and reflects the broader secondary outcomes observed in the main trial, such as improved voice understanding and empowerment [9].

Although use of technology initially caused some anxiety, its role in externally representing voices was well accepted by most, mirroring AVATAR1 qualitative findings [11]. Participant accounts regarding their emotional connection to, or disconnection from, the avatar relate to the concept of sense of voice presence [12,13]. This is a key mechanism in fear activation [43], promoting emotional processing and generalization of learning [44,45]. In line with earlier work [13], most participants reported at least some degree of voice presence during avatar dialogues. However, early challenges involved either high levels of perceived realism leading to fear activation (particularly in the context of safety behaviors, especially avoidance, and reactivation of trauma memories) or difficulties accessing voice presence inhibiting emotional processes. This reflects the bidirectional relationship between presence and emotion [46]. Participant accounts support the assertion that engagement and positive outcomes are contingent on voices being brought online within dialogues to activate emotion (ie, anxiety) within a safe therapeutic space [13].

Some participants indicated that, in the context of a high sense of voice presence, they experienced temporary symptom exacerbation. However, consistent with previous findings [13], the emotional intensity of this experience appeared well-tolerated by most and promptly eased across therapy. In fact, many specifically valued coming through this experience because it meant they had directly confronted and overcome core issues. This is consistent with evidence of temporary symptom exacerbation when working through challenges in trauma-focused imaginal exposure [47] and other exposure therapies [48,49].

Participants identified how open-minded attitudes, resilience, and determination helped to overcome the challenge of direct work with voice content, particularly when navigating anxiety-provoking early dialogues. While often conflated with attendance [50], many emphasized that engagement went beyond simply turning up each week and involved “leaning into” therapy to access voice presence, emotional processes, and meaningful outcomes. The emotional experience of therapy ending was also highly individualized across both AV-BRF and AV-EXT, with some experiencing a profound sense of closure and others struggling with loss. This variability reflects individual differences and the diverse meanings and relationships involved in voice-hearing, highlighting that the experience of therapy ending can be shaped by personal meaning and relational factors [51].

Generalizing learning from avatar dialogues to voices in daily life could be challenging, reflecting broader literature and emphasizing the need for continued use of skills [52]. The 3 therapy completers did not experience notable positive outcomes, possibly reflecting reported challenges in accessing voice presence, as this has been linked to the transfer of knowledge and skills [13].

It is important to note that participants who discontinued therapy were underanalyzed in relation to voice presence, with only 1 included in the theme “Collaborative efforts facilitated meaningful connection to avatar.” The 2 other participants disengaged prior to any dialogues in the context of challenges with the realism of the software, meaning they did not reach the point of establishing presence. These issues are further considered in relation to dropout experiences below.

### How Was TA Experienced by Participants in the Context of AVATAR Therapy?

Participant accounts align with the concept of TA [53], emphasizing therapist qualities like warmth and empathy. Strong TA was established for all, including those who dropped out, underscoring its central role in therapy experience, engagement, and outcomes, consistent with other psychosis interventions [54-56]. Developing trust was perceived to be vital given sensitive therapy processes, such as sharing verbatim voice content, exposure to feared stimuli, and working with trauma. In particular, participants valued transparent, respectful conversations to ensure an appropriate balance of voice presence and safety, particularly when reenacting abuse via the avatar [8]. Therefore, TA could mitigate the impact of the therapist operating the avatar and facilitate continued engagement despite anxiety. Therapists’ emotional attunement within avatar dialogues enabled person-centered therapeutic input, which progressively reduced across therapy [40].

Despite initial concerns about the impact of technology on TA, most participants felt they had adequate access to human therapist support. In fact, the digitally mediated environment was perceived to provide therapists with unique insights into participants’ live experiences and behaviors, creating valuable opportunities for validation. Participants also valued therapists’ respectful curiosity about their experiences, values, beliefs, strengths, and cultural background. In this way, many described feeling empowered to take an active role in their therapy, emphasizing collaboration and choice [57,58].

### What Were the Experiences of Participants Who Decided Not to Continue With Therapy?

Choosing to discontinue therapy could be an empowering choice, providing insights for recovery, but could also be experienced negatively, reflecting the contrasting experiences of therapy dropout [59]. This also emphasizes that attendance is only one component of engagement, and it is possible to meaningfully access therapeutic processes without reaching a treatment dose [50].

While challenges of exposure to the avatar eased promptly for most, the emotional toll of dialogues across therapy led 1 participant to drop out after 5 dialogues (8 sessions). Additionally, 2 other participants chose to discontinue prior to any dialogues due to difficulties with the realism of the avatar software. This suggests that AVATAR therapy may be overwhelming if voice-hearing experiences elicit high levels of anxiety [60], and, conversely, insufficient realism may disrupt voice presence and engagement. In this way, both too much and too little realism can create difficulties in establishing voice presence and activating target emotions in a safe setting, posing a risk for disengagement. However, many therapy completers who benefited from AVATAR therapy reported similar challenges in early sessions and initially questioned whether to continue, highlighting the role of personal choice. Factors such as life events outside of therapy, access to resources, and social functioning may influence an individual’s decision to continue with therapy despite challenges [61-63]. This was endorsed by therapy completers who acknowledged that AVATAR therapy may not be effective for all, depending on factors such as stage in recovery, stressful life events, and physical health, consistent with wider literature [47,64-66]. Taken together, this indicates that dropout may not simply reflect limitations in software realism but also readiness for exposure and suitability of the intervention for the individual at that time.

Similarly to AVATAR1 qualitative findings [11], 1 autistic participant struggled to engage with the idea of digitally embodying the voice, possibly suggesting additional challenges for those with this comorbidity. This highlights the need for further research specifically focused on autistic voice-hearers.

### Strengths and Limitations

The study demonstrated methodological rigor with adherence to peer research methods and IPA principles, such as using a phenomenological approach, ensuring reflexivity, and using an iterative process of analysis [18]. Steps were taken to standardize interview delivery through training, role-play, co-interviewers, and supervision. The peer researcher status of interviewers likely supported participants to feel comfortable, understood, and willing to open up [67]. Additionally, efforts were made to include participants who discontinued AVATAR therapy, enhancing learning around potential reasons for making this decision. 

While integrating 2 qualitative analysis methods enabled both depth and breadth [36], this introduced epistemological tension as IPA emphasizes idiographic depth while template analysis prioritizes cross-case patterns. The careful layering of these methods was guided by advice from experts in qualitative research methods and supported by reflexive practices and peer research methods. However, some complexity may have been reduced when moving to broader themes, with the risk of diluting the nuance of individual voices. This highlights the trade-off between depth and generalizability [21].

Selecting transcripts for IPA based on richness of data (often defined in terms of depth, detail, and context provided within narratives) has been questioned due to the associated risk of bias in privileging some accounts over others [22]. In line with IPA principles, transcripts of participants who discontinued therapy were not included in IPA to ensure homogeneity [19,34]. While the integration of template analysis enabled clear comparison of completers and noncompleters and ensured that all participants’ perspectives were systematically incorporated [23], this may have privileged voices that were more positive and engaged. This could limit the transferability of findings, as the therapeutic processes identified in detail may not fully reflect the experiences of those who disengaged or found the intervention unsuitable.

Participants were invited for an interview after completing the 28-week follow-up to avoid any influence on trial outcomes. Therefore, interviews were conducted several months after completing AVATAR therapy, enabling exploration of long-term perceived impacts. However, this delay may have also introduced recall bias or retrospective reinterpretation of participants’ experiences during therapy. Social desirability bias and self-selection bias may have impacted study recruitment. While we recruited a broadly representative sample with increased numbers of participants who dropped out of therapy compared to AVATAR1, only 4.3% (3/69) of those who dropped out were represented compared to 9.9% (16/161) of therapy completers. As it was not possible to recruit the intended 4 participants who dropped out, and the interview sample included 2 participants who dropped out prior to any dialogues, we have likely missed valuable experiences and perspectives from those who were not willing to participate. Additionally, as this study focused on TA between the voice-hearer and therapist, we may have missed important insights into the “triangle of alliance” between the participant, therapist, and digital platform (ie, avatar) [68].

A total of 10.5% (2/19) of participants were from Black backgrounds compared to 17.8% (41/230) in the wider AVATAR2 sample. The experiences of Black participants are the focus of a stand-alone qualitative study within the AVATAR2 Programme of Qualitative Research, which is currently being prepared for publication [69]. Concomitant recruitment for both qualitative studies is a likely factor in the lower representation of Black participants within this study.

### Implications for Optimization and Implementation

The AVATAR2 trial aimed to evaluate the efficacy of 2 forms of AVATAR therapy, determine optimal therapy delivery, and consider implementation in National Health Service (NHS) settings. Supported by a National Institute for Health and Care Excellence Early Value Assessment [70], which has recently recommended further testing in routine NHS settings, these qualitative findings will support the optimization and wider implementation of this approach.

Establishing voice presence and activating target emotions within a safe setting has been emphasized as central to engagement and outcomes. Software developments may improve the realism of digital representation, enhancing sense of voice presence. Findings emphasize the need for improvements to the tailoring of avatars across different ethnicities and genders to ensure equal opportunities to establish voice presence. However, as technology advances, increased realism might activate strong emotional responses and increase fear within the digital environment, particularly pertinent given the ongoing trial of AVATAR-VR [71]. Therefore, flexible therapist support will be crucial to ensure participants feel safe, balancing exposure with tolerability [8,47]. Meanwhile, dropout experiences suggest that difficulties establishing presence may not only reflect technological limitations in software realism but also individual readiness and suitability, emphasizing the importance of personalizing AVATAR therapy to the person’s stage of recovery and capacity to tolerate exposure.

Limitations suggest that our findings are most transferable for understanding therapeutic processes when engagement is maintained, providing less insight into disengagement and nonresponse. Therefore, future work should further explore the perspectives of those who discontinue or gain limited benefit. Such insights could inform early screening and tailoring*,* for example, around readiness for exposure and tolerance of voice presence.

In this context, integrating emotion regulation and anxiety management techniques may enhance coping and sense of safety [47,72], as demonstrated by Paulik et al [73] in supporting voice-hearers to engage with exposure therapies. Therefore, future research could consider if an emotion regulation phase improves the tolerance of this experience, ensuring trauma-informed care and integrating other therapeutic approaches [47]. Additionally, tools to support normalizing discussions around common voice content and transparency over potential temporary voice exacerbation could facilitate collaborative engagement and reduce distress.

Findings highlight the importance of TA, fully informed consent, and patient choice when proceeding with exposure and dialogues. Peer support spaces could provide additional support, increase motivation, and promote engagement and outcomes [74,75]. Further research could explore the triadic relationship between the participant, therapist, and avatar. In particular, it would be valuable to examine how the concept of TA applies to the avatar, given its role in representing the distressing voice yet also the individualized relational context, meaning that there may be a therapeutic focus on fostering compassion toward the voice [8,76].

The range of emotional experiences across AV-BRF and AV-EXT highlights the complexities and relevance of individual differences. AVATAR2 RCT findings suggest that AV-EXT produces stronger results, but this was in the context of higher dropout rates. As AVATAR therapy moves from clinical trials and into routine care, there are increased opportunities for tailoring the therapy to context, including flexibility in duration, use of booster sessions, and potential integration into long-term therapy.

### Conclusions

This study provides valuable insights into the participant experience of AVATAR therapy, enriched by peer research methods. Overall, experiences were positive, with all reporting strong TA and most gaining power over the avatar and, consequently, voices. There were challenges with in vivo exposure and dialoguing with voice content, but this was often mitigated by TA. Engagement and positive outcomes were contingent on establishing voice presence and activating target emotions while retaining a sense of safety. Potential barriers to engagement and outcome have been suggested, emphasizing the need for software developments, person-centered flexibility, and informed choice.

## Appendix

Multimedia Appendix 1 Stages of PPI.

Multimedia Appendix 2 Interview guide.

Multimedia Appendix 3 Interview training materials.

Multimedia Appendix 4 Excerpts of analytic processes.

Multimedia Appendix 5 Provisional template.

Multimedia Appendix 6 Summary of themes.

Multimedia Appendix 7 Additional quotes.

## Abbreviations

AV-BRF: AVATAR-Brief, a 6-session version of AVATAR therapy
AV-EXT: extended (12-session) form of AVATAR therapy
IPA: interpretative phenomenological analysis
NHS: National Health Service
PPI: patient and public involvement
RCT: randomized controlled trial
TA: therapeutic alliance
UCL: University College London

## Data Availability

This study was preregistered in the Open Science Framework [30]. Although anonymized, the qualitative data generated for this study are potentially identifiable, and participants only provided written informed consent for anonymized sections to be published. Therefore, the qualitative datasets are not suitable to be deposited in a public database. However, the data are available from the corresponding author on reasonable request.
