# Supplementary figures and images for: “It Felt Good to Be Able to Say That Out Loud”—Therapeutic Alliance and Processes in AVATAR Therapy for People Who Hear Distressing Voices: Peer-Led Qualitative Study
Source: JMIR Ment Health. 2026 Jan 28;13:e77566. doi: 10.2196/77566 (PMC12895157; doi:10.2196/77566)

Supplementary material 1: Stages of PPI

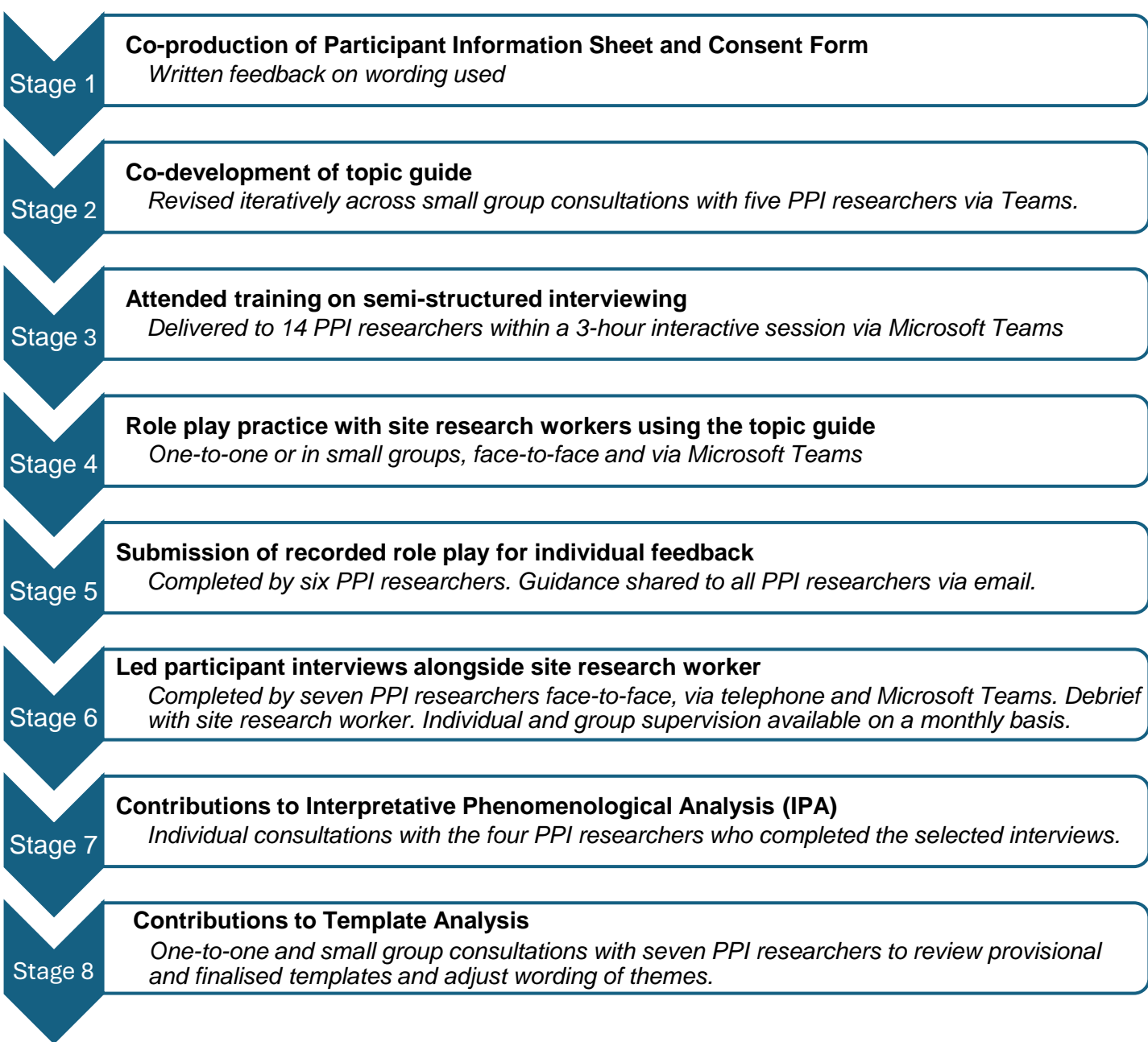

Supplement: Multimedia Appendix 1 [file mental_v13i1e77566_app1.pdf]

**Supplementary material 5 – Provisional ‘template’**


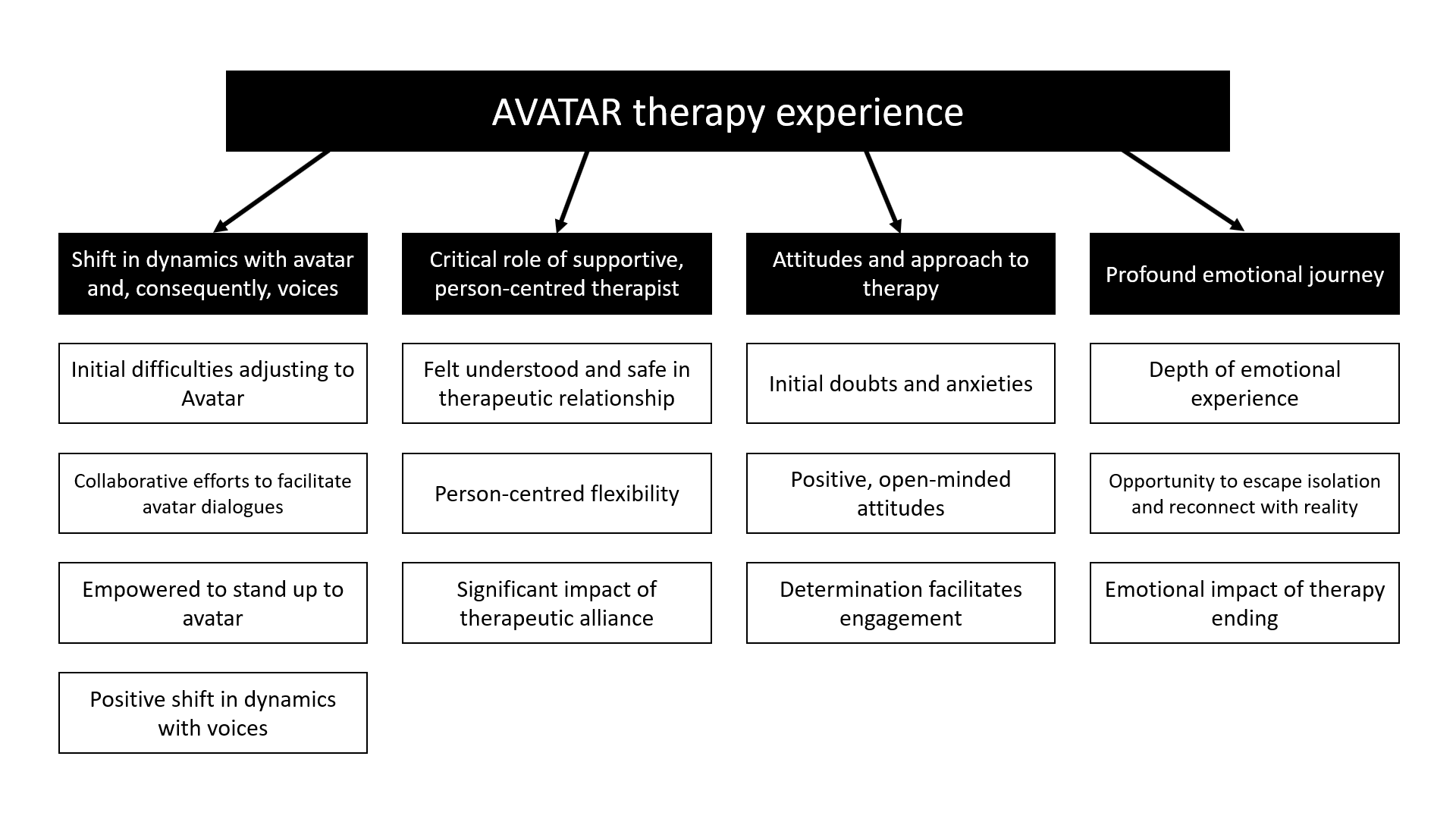

Supplement: Multimedia Appendix 5 [file mental_v13i1e77566_app5.docx]
