# Supplementary material for: “It Felt Good to Be Able to Say That Out Loud”—Therapeutic Alliance and Processes in AVATAR Therapy for People Who Hear Distressing Voices: Peer-Led Qualitative Study
Source: JMIR Ment Health. 2026 Jan 28;13:e77566. doi: 10.2196/77566 (PMC12895157; doi:10.2196/77566)
Supplement: Multimedia Appendix 2 [file mental_v13i1e77566_app2.docx]

**Supplementary material 3: Interview topic guide**

| **Introduction** |
| --- |
| *Introductions – Names, institution & NHS trust, roles. If PPI member has previously been involved with AVATAR therapy / research, they could share this if they feel comfortable.*  Thank you for agreeing to speak with me today.  Today is not a therapy session but we will be asking you some questions about your experiences of receiving AVATAR therapy.  This interview will take no longer than an hour to complete.  If you have any questions at any point, please do not hesitate to ask. |
| **Confidentiality and consent:** |
| Thank you for having taken the time to read through the information sheet and consent form we sent to you.  *Ask if they have any questions.*  *Ask if they are willing to sign the consent form.* |
| **Introductory questions / Getting involved in AVATAR2** |
| *We are first going to think back to when you first heard about AVATAR therapy and how you have found it.*  How did you first hear about AVATAR therapy?  How did you feel about receiving AVATAR therapy?  How have you found AVATAR therapy? |
| **Experience of working with the avatar.** |
| *We’re now going to spend a bit of time talking through what it was like to work directly with the content of the voice you hear through the avatar.*  What was it like to share the content of the voice with your therapist?  What was it like to hear the words the voice says through the avatar in this different way?  What was it like to have a dialogue with the avatar? |
| **The therapeutic relationship in the context of AVATAR therapy** |
| *We will* *now spend some time talking about the relationship with your therapist.*  What was your experience with your therapist like?  Have you had therapy before? Was the relationship with your therapist different to previous therapeutic relationships?  What was it like to experience your therapist operating the avatar to match your voice? |
| **Understand reasons for early drop-out (if relevant)** |
| Did you take part in all of your sessions?   - **If not**, when did you decide to stop?   Why did you decide not to continue with therapy?   - **If so**, what kept you going?   Did you ever consider stopping?  What would you say to someone who was thinking about taking part in the therapy? |
| **Ending** |
| Is there anything else you wanted to say about your experience of AVATAR therapy that we have not discussed?  Thank you for taking part in this interview today.  There is information about different phone numbers and services you can reach out to if you need additional support.  Do you have any questions? Please feel free to get in touch – The contact details are on the information sheet.  What are you planning on doing for the rest of the day? |
| **Notes – Please note down any comments on the participant’s presentation during the interview e.g. if they appeared upset or distressed.** |
