# Supplementary material for: “It Felt Good to Be Able to Say That Out Loud”—Therapeutic Alliance and Processes in AVATAR Therapy for People Who Hear Distressing Voices: Peer-Led Qualitative Study
Source: JMIR Ment Health. 2026 Jan 28;13:e77566. doi: 10.2196/77566 (PMC12895157; doi:10.2196/77566)
Supplement: Multimedia Appendix 3 [file mental_v13i1e77566_app3.pdf]

AVATAR2 INTERVIEW TRAINING

Part 1

1

AGENDA

- Setting up
- Safeguarding and confidentiality
- Disclosure of your own experiences
- What will happen on the day
- Support and supervision

2

SETTING UP

- Introduce yourself and role.
- Check purpose of the meeting is clear. (Info sheet 24hrs before).
- Clear expectations – about 1hr, can take breaks, stop any time, confidential space (more on that later), recording.
- Informed consent – RA to lead on this.
- Interview begins.

SAFEGUARDING AND CONFIDENTIALITY

- Confidentiality

→ Important for participants to know what will happen with the information we get from the interviews. Reminder of key information: e.g. Interviews will be audio-recorded – all recordings to be deleted once we have transcribed them.

- Risk

→ If you're interviewing service users, someone may tell you something that suggests some risk of harm to themselves or someone else.

→ Risk can take many forms; self-neglect,

This information must be shared with clinical team, and the person supported to access some help – Research Assistants are trained in these protocols, trust your instincts and talk to them if you have any worries about someone.

DISCLOSING YOUR OWN EXPERIENCES

One important aspect of being a peer researcher is that it involves disclosing that you have lived experience of mental health difficulties and have used mental health services – for unusual distressing experiences.

DISCUSSION: What would you like others to know about your identity? / Pros and cons of sharing your own lived experiences?

DISCLOSING YOUR OWN EXPERIENCES

PROS

- Service-users involved in the research might feel that you understand what they're going through.
- Service users involved in the research might trust you and be honest with you about their views and feel less worried about being judged.
- Other benefits?

CONS

- Consider how much are you comfortable to disclose
- Avoid disclosing things that are too personal or distressing
- Avoid too much depth
- Avoid overly influencing the research participant
- Other considerations?

WHAT WILL HAPPEN ON THE DAY

- Brief - Before every interview, we can meet to discuss the plan for the interview and any concerns/questions you may have.
- Interview - What role would you like to have? Are there parts of the interview that you would particularly like to do?
- Debrief - After every interview, we can meet and speak about any questions, or debrief if you found anything particularly difficult in the interview.

SUPPORT AND SUPERVISION

- What will happen if need extra support on the day.
- Monthly peer supervision: Learning, ongoing reflections and peer support.
- Monthly drop-in supervision: One-to-one support as required.
- Contact local trial coordinators for urgent queries: Provide contact details
- Contact your local Research Assistant for less urgent enquires

THANK YOU

15-minute break

AVATAR2 INTERVIEW TRAINING

Part 2

AGENDA

- Introduction to interview skills
- Open questions
- Empathy and validation
- Demonstration and role play
- Summary

INTRODUCTION TO INTERVIEW SKILLS

It is important to build rapport with the person and try to make them feel comfortable speaking with us.

Therefore, it is helpful to be familiar with key interview skills, such as active listening, open questions and validating.

OPEN VS. CLOSED QUESTIONS

What is the difference between open and closed questions?

→ Open questions are questions that invite someone to answer a question in their own words and provide as much detail as they wish.

→ Closed questions can be answered with "Yes" or "No," or have a limited set of possible answers.

Open questions may start with "How" or with words that begin with "w" such as "What", "When", "Why", "Where", "Which" and "Who"?

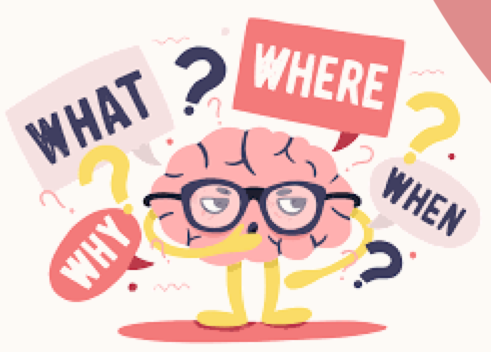

13

OPEN VS. CLOSED QUESTIONS

For example:

→ CLOSED: Did you feel able to trust your therapist?

→ OPEN: How did you feel about your therapist?

DEMONSTRATION – Open vs closed questions

What did you notice?

Any thoughts or reflections?

14

OPEN QUESTIONS

PROS:
- Allow for unlimited responses
- Provide more detail

CONS:
- Time-consuming to answer
- A lot of irrelevant information

Both open and closed questions are important, but we don't want to ask too many closed questions.

15

EMPATHY

What is empathy?

Brené Brown on Empathy vs Sympathy - YouTube

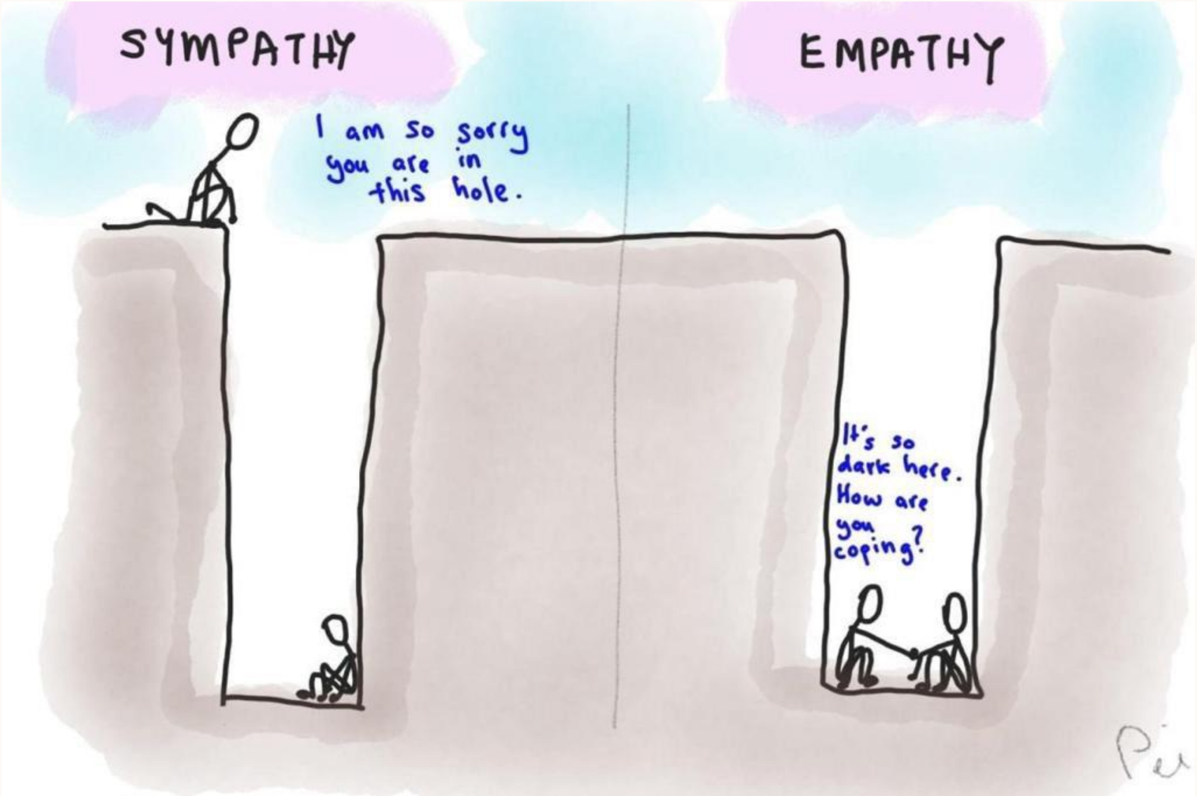

16

EXAMPLES OF EMPATHY

- "From what you've told me, it sounds like the situation was incredibly difficult for you"
- "That must have been really stressful for you"
- "I can imagine how frustrating that would be"
- "I can totally see why you would be upset"

ACTIVE LISTENING AND VALIDATION

Show you're listening

→ Eye contact, nodding, facial expressions, etc.

→ Repeat back to the person what they've said (briefly) – This is useful to check you've got it right, and to encourage them to say more.

→ Use the person's language when summarising:

- e.g. 'when you were feeling all over the place...'

EXAMPLES OF VALIDATION

- "Anyone in that situation would have come to a similar conclusion"
- "Given what you've been through, it's completely understandable how that could have felt triggering".
- "I can see why it may have been difficult to respond to the questions when you were experiencing a lot of voices"

ROLE PLAY

Tell me about a time you were running late OR got lost.

Exercise: Use empathy, validation and active listening, and a mixture of open and closed questions, to find out more about their experiences of running late / getting lost.

Interviewer, Interviewee, Observer – 5 mins then swap roles.

1 person to feedback to wider group – Something that went well and something that was more difficult.

AVATAR2 Interview training 21

SUMMARY

- ❖ Mixture of open and closed questions
- ❖ Empathy
- ❖ Active listening
- ❖ Validation

21

THANK YOU

15-minute break

22

AVATAR2 INTERVIEW TRAINING

Part 3

AGENDA

- Introduction to the topic guide
- Practice sections of the interview
- Final top tips
- Next steps
- Questions

INTRODUCTION TO TOPIC GUIDE

This is a guide of key questions to ask participants in the interview:

→ The guide gives topic areas that we would like to discuss and suggests questions / prompts.

→ The structure of the interview isn't rigid, and it is not a script or questionnaire but is there to guide and support engagement.

We are trying to get a better understanding of AVATAR2 participants' experiences of AVATAR therapy:

- ❖ Experiences of working directly with the words the voice(s) say.
- ❖ Relationship with their therapist.
- ❖ Reasons for not completing therapy.

ROLE PLAY 1

What was it like to challenge the voice?

Potential extra prompts:

- Was it an emotive or scary experience?
- Did the avatar feel real?
- Did you feel any reluctance to engage with the voice?

Exercise: Ask these and any other questions that come to mind to find out more about the experience of working with the voice in AVATAR therapy.

Interviewer, Interviewee, Observer – 5 mins then swap roles.
1 person to feedback to wider group – Something that went well and something that was more difficult.

ROLE PLAY 2

What was your experience with your therapist like?

Potential extra prompts:
- Did you feel able to trust your therapist?
- Did you feel that the therapist understood your experiences?
- What did the therapist do that helped you feel supported?

Exercise: Ask these and any other questions that come to mind to find out more about the experience of the therapeutic relationship in AVATAR therapy.

Interviewer, Interviewee, Observer – 5 mins then swap roles.
1 person to feedback to wider group - Something that went well and something that was more difficult.

FINAL TOP TIPS

It is okay to pause for a few minutes, slow down or hand over to the other interviewer – "Just give me a moment please"

Even when you ask closed questions, you can ask an open question at the end, such as "What else would you like to say about that?"

Remember to ask the person how they are doing throughout the interview – "How are you doing?", "Would you like a break?"

NEXT STEPS

LET US KNOW	DBS CHECK	PRACTICE	RECRUITMENT	AVAILABILITY
Let us know if you would like to take part in delivering the interviews.	We can then arrange DBS checks to be completed before interviews start.	Arrange practice sessions with your local Research Assistants and other PPI members.	We will begin contacting AVATAR2 participants to invite them to be interviewed.	Let us know your availability and preferences for face-to-face or online. We will then contact you to arrange interview dates!

29

THANK YOU

Any questions?

30
