# Supplementary material for: “It Felt Good to Be Able to Say That Out Loud”—Therapeutic Alliance and Processes in AVATAR Therapy for People Who Hear Distressing Voices: Peer-Led Qualitative Study
Source: JMIR Ment Health. 2026 Jan 28;13:e77566. doi: 10.2196/77566 (PMC12895157; doi:10.2196/77566)

**Supplementary material 4: Excerpts of analytic processes**


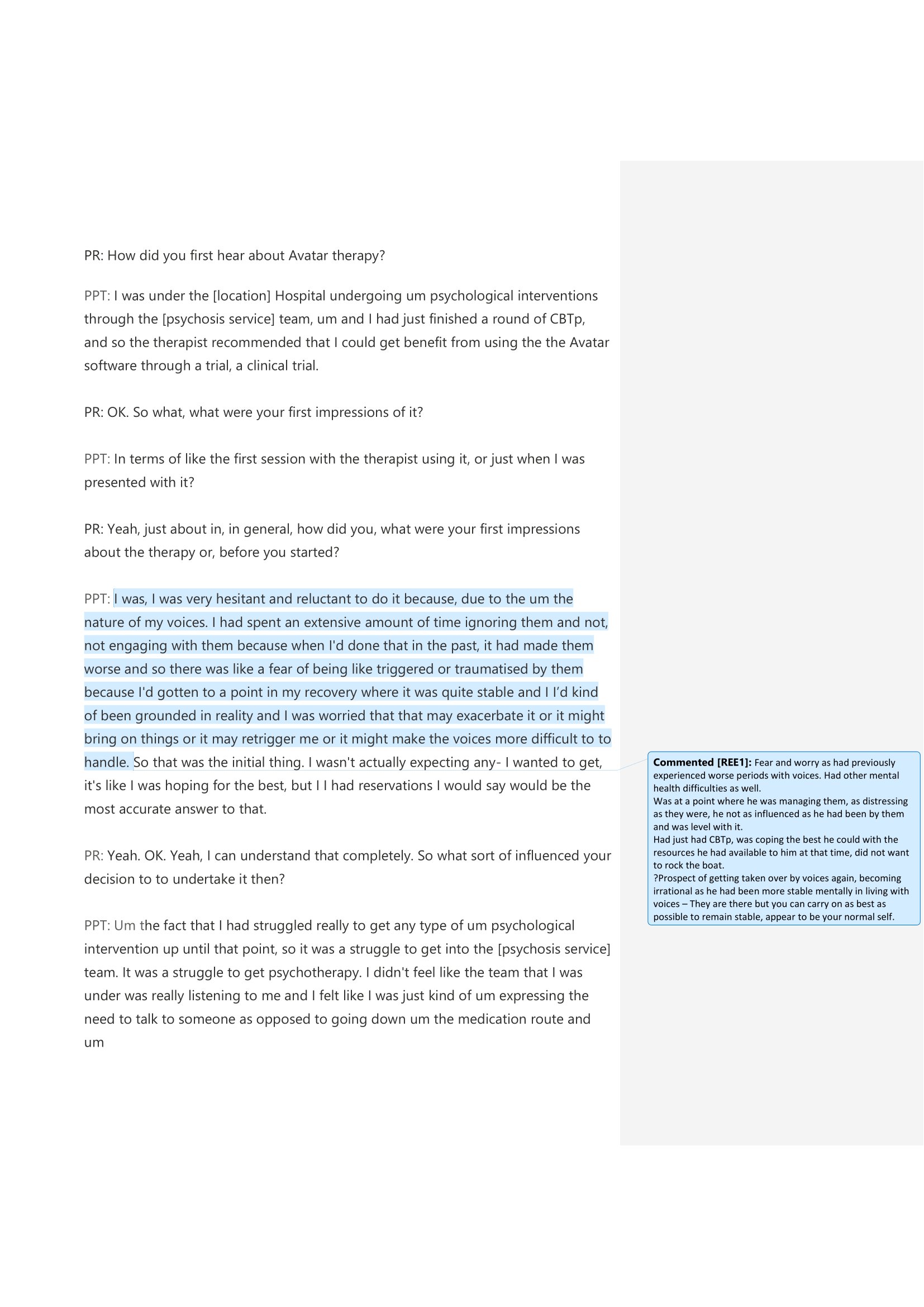
Peer researcher perspectives – Added as comments to transcript in their own words

Transcript with exploratory noting


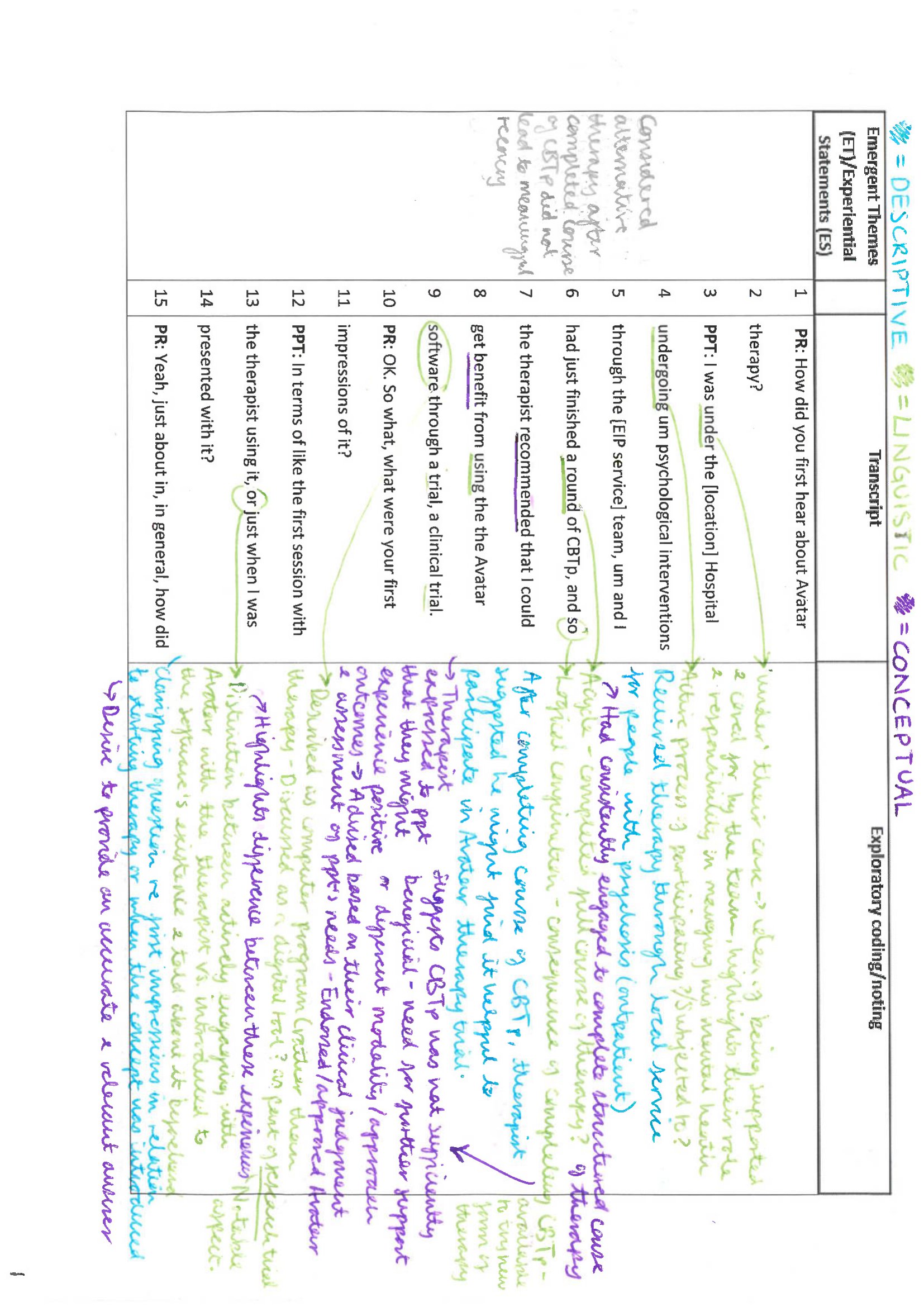


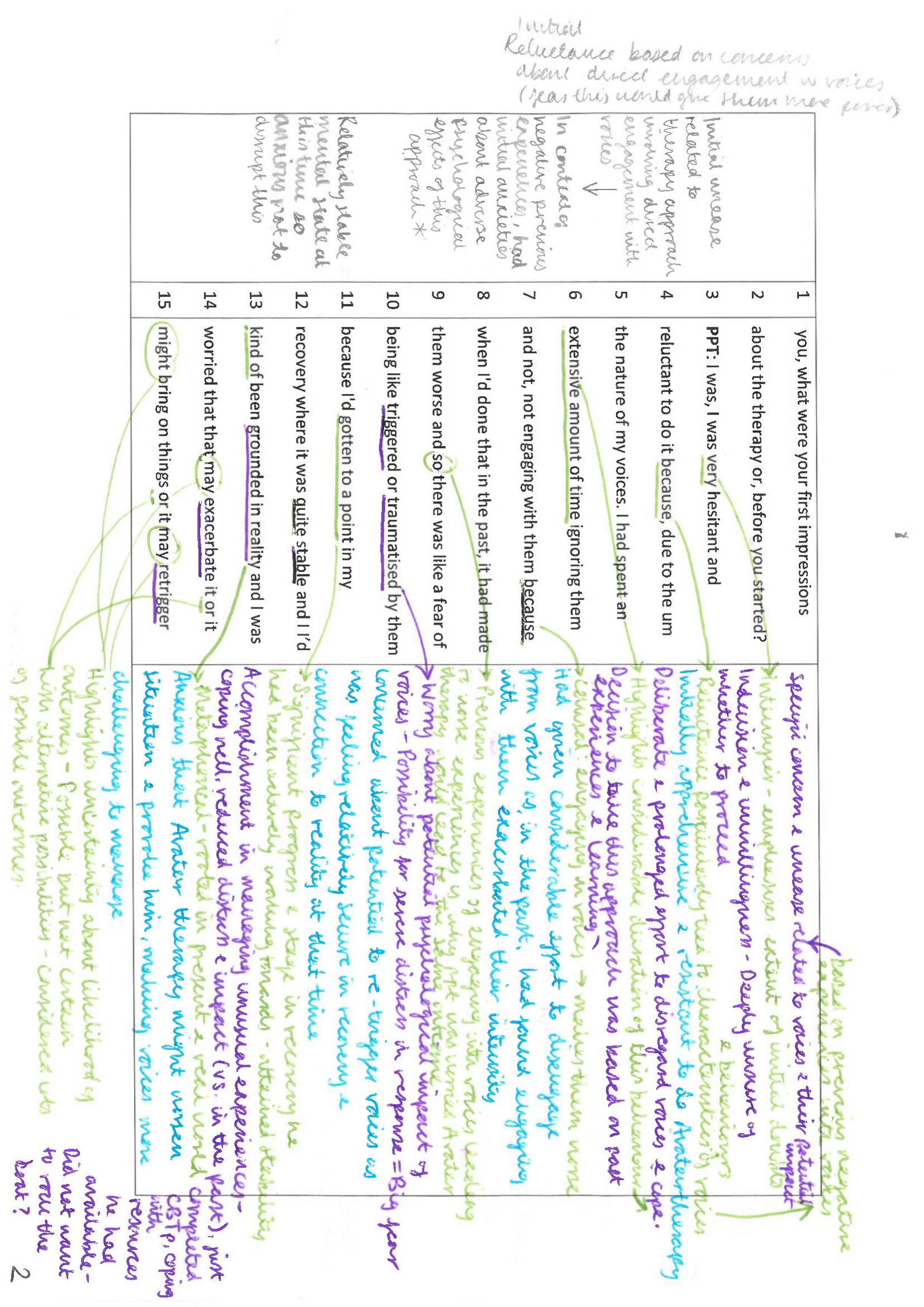


Visual arrangement of experiential statements to demonstrate PETs and subthemes.


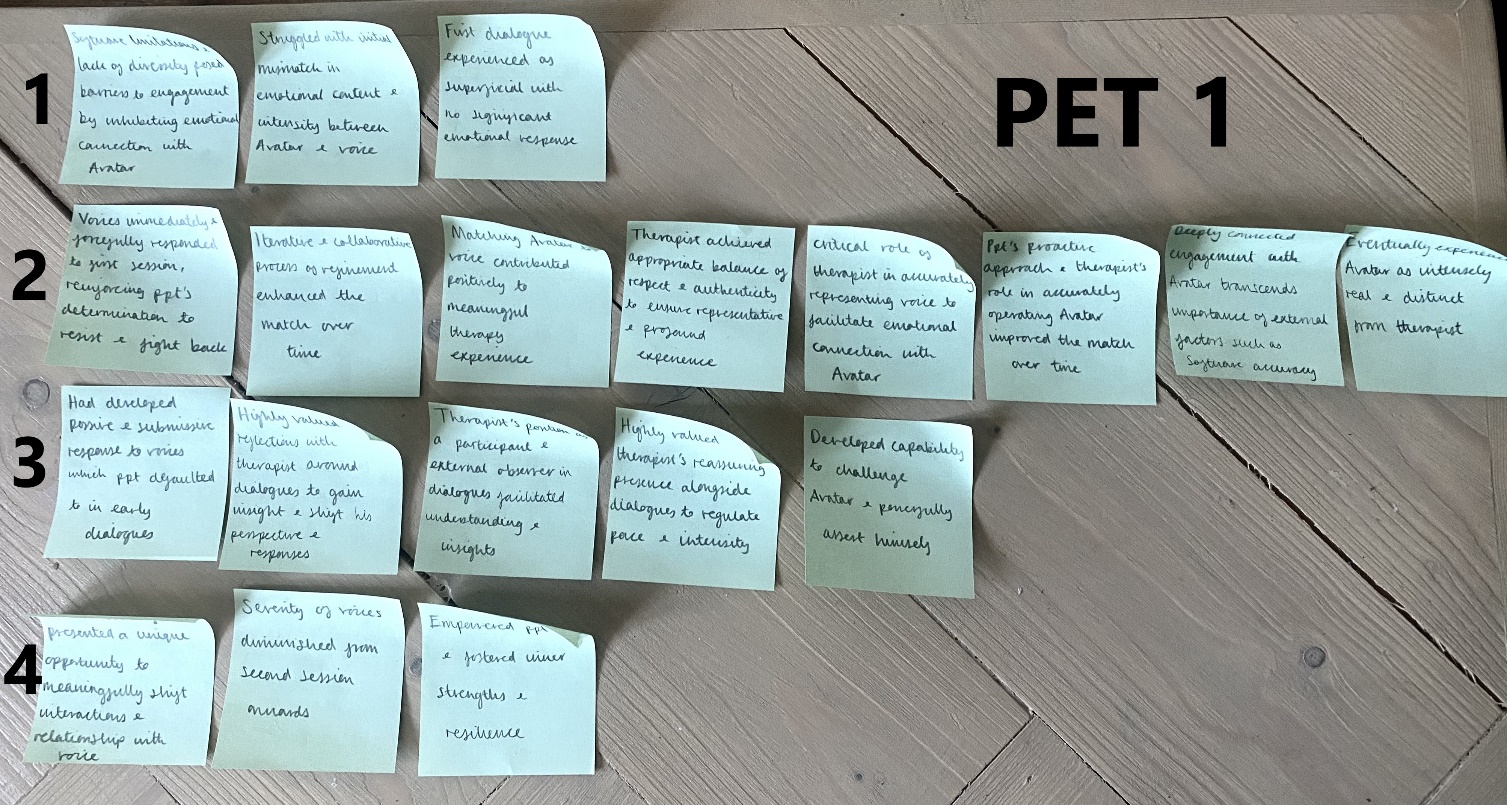


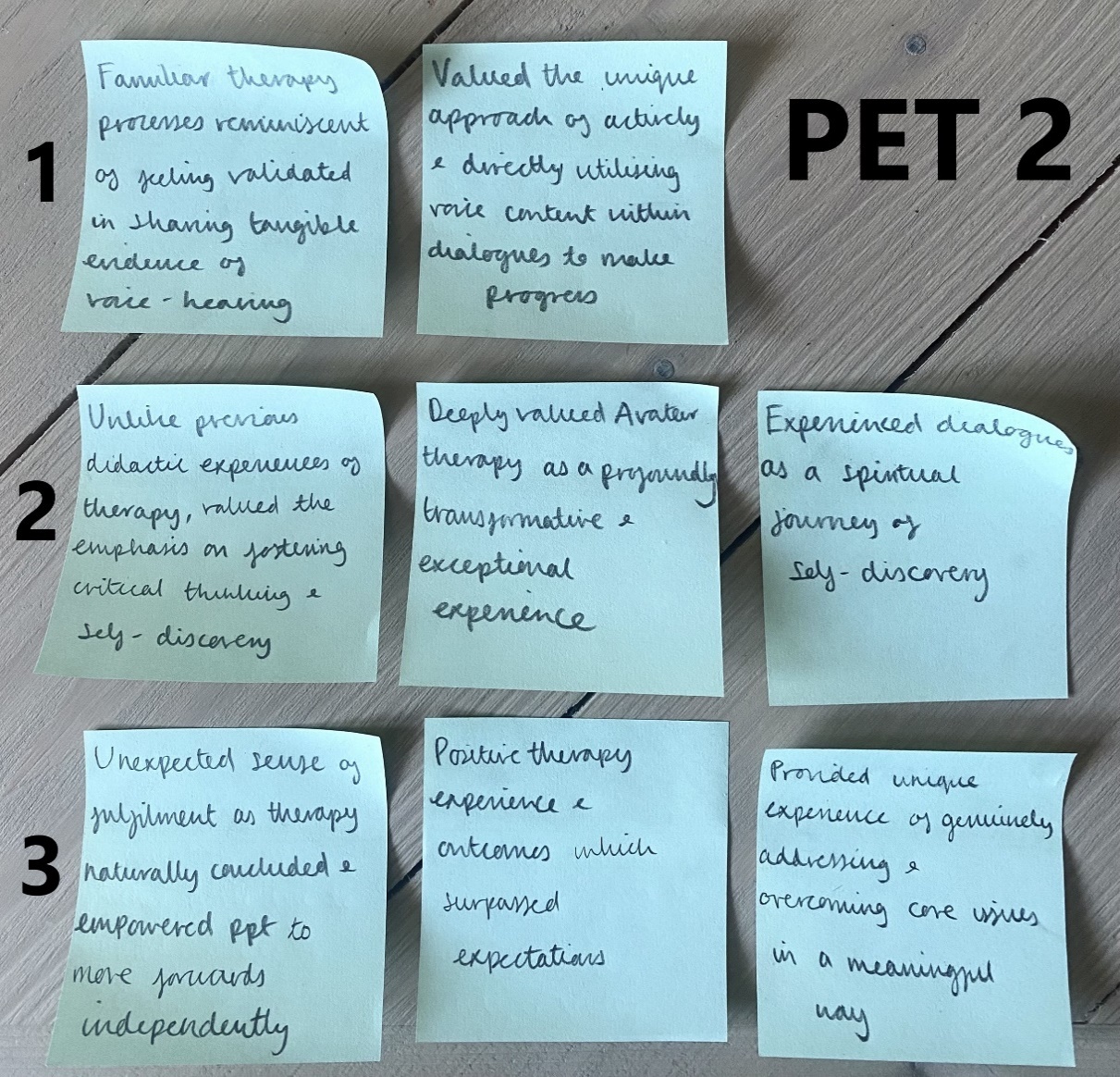


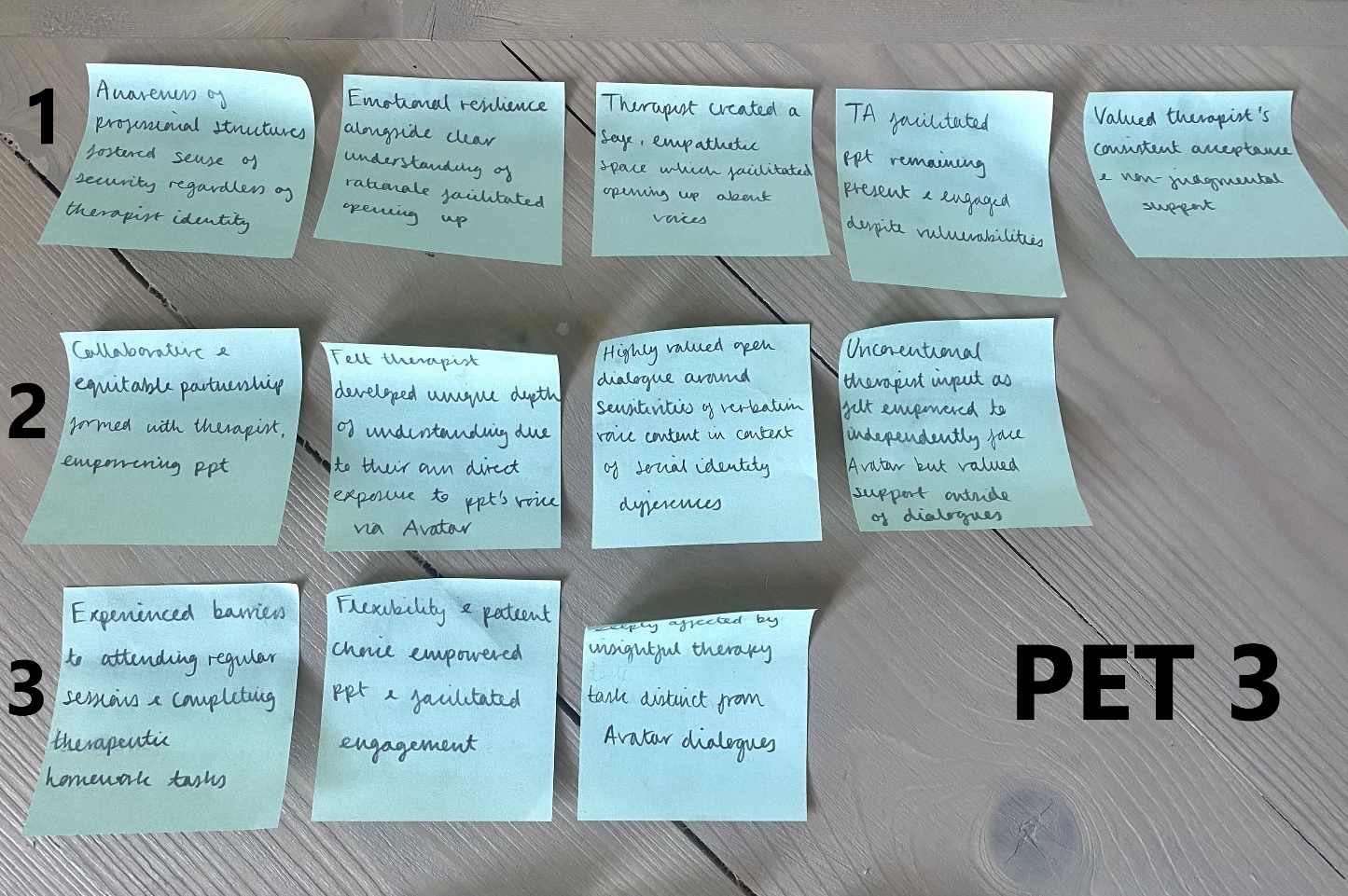


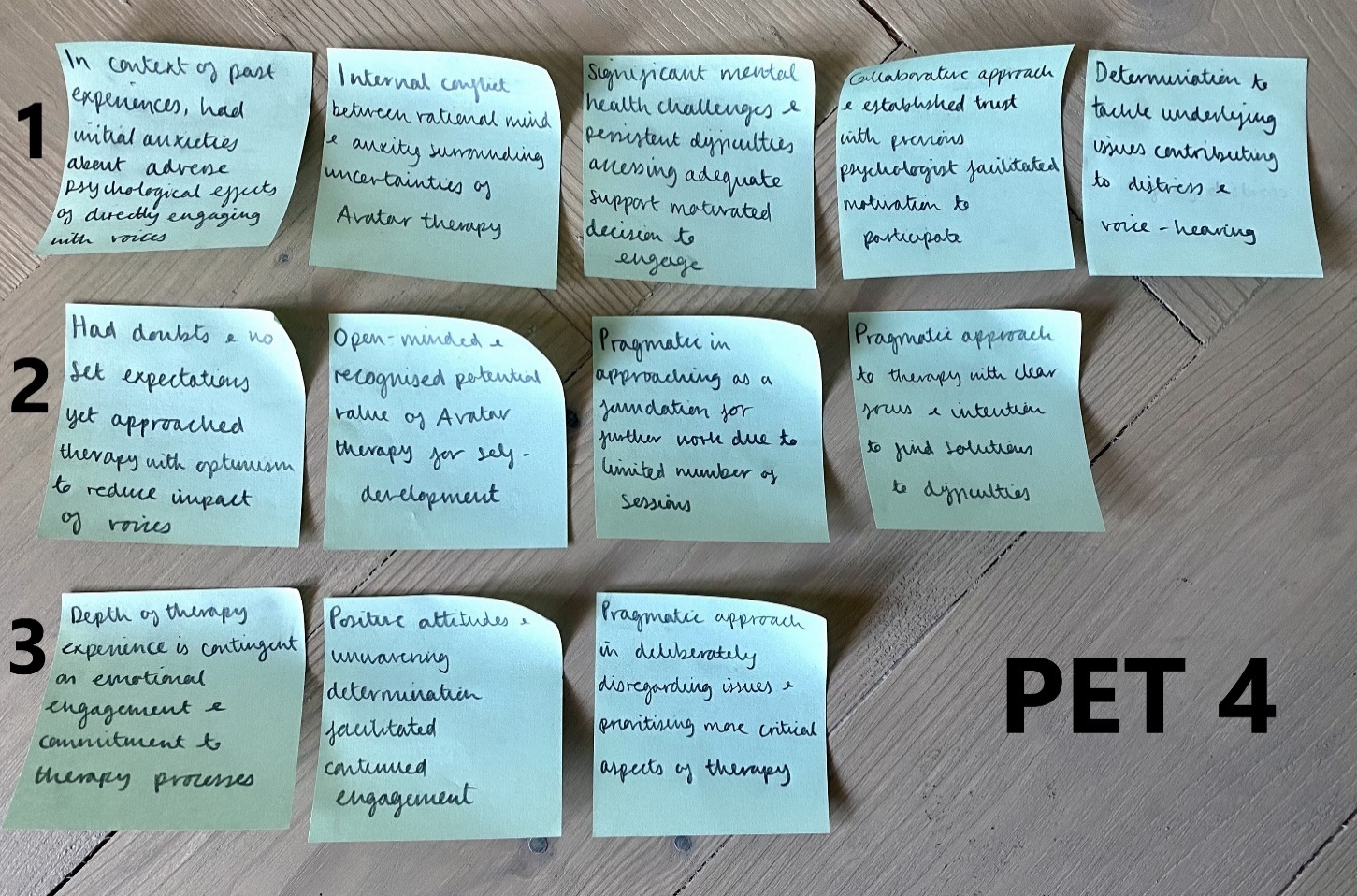


Final structure of Personal Experiential Themes (PETs) and subthemes


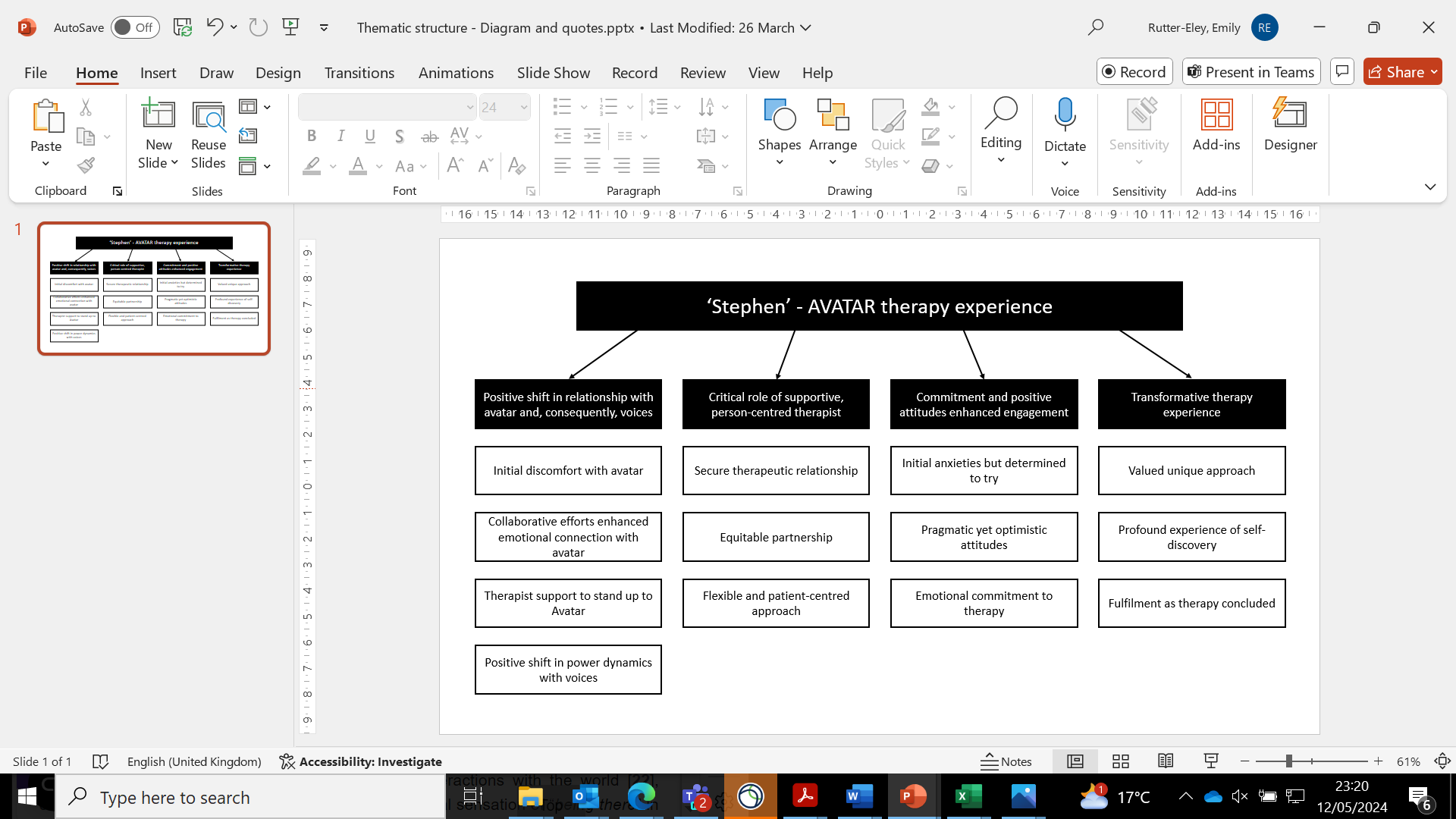

Supplement: Multimedia Appendix 4 [file mental_v13i1e77566_app4.docx]
