# Supplementary material for: “It Felt Good to Be Able to Say That Out Loud”—Therapeutic Alliance and Processes in AVATAR Therapy for People Who Hear Distressing Voices: Peer-Led Qualitative Study
Source: JMIR Ment Health. 2026 Jan 28;13:e77566. doi: 10.2196/77566 (PMC12895157; doi:10.2196/77566)
Supplement: Multimedia Appendix 6 [file mental_v13i1e77566_app6.docx]

**Supplementary material 6: Summary of themes**

| Overview of themes | Overview of subthemes | n (%) of sample | n (%) of completers | n (%) of non-completers |
| --- | --- | --- | --- | --- |
| Shift in relationship with avatar and, consequently, voices: Exposure and in vivo communication with verbatim voice content via avatar and subsequent changes with voices | ***Initial challenges adjusting to avatar****:* The broad range of experiences, including anxiety, discomfort and feeling emotionally disconnected | 18 (94.7%) | 15 (93.8%) | 3 (100%) |
|  | ***Collaborative efforts facilitated meaningful connection to avatar****:* Participant efforts and therapist support enhanced adjustment and connection to the avatar | 14 (73.7%) | 13 (81.3%) | 1 (33.3%) |
|  | ***With therapist support, participants felt empowered to stand up to avatar****:* Gradual but distinct shift from overpowered to empowered across dialogues | 16 (84.2%) | 15 (93.8%) | 1 (33.3%) |
|  | ***Positive shift with voices****:* Changes in voice frequency, omnipotence and interactions, as well as understanding, coping and perspectives | 15 (78.9%) | 13 (81.3%) | 2 (66.7%) |
| Crucial role of person-centred therapist: Valued therapist qualities and the impact of therapeutic alliance on engagement, experience and outcomes | ***Felt safe, supported and understood****:* Professional structures and therapeutic qualities fostered a safe, supportive space | 19 (100%) | 16 (100%) | 3 (100%) |
|  | ***Person-centred flexibility****:* Collaborative and individualised approach empowered participants to meaningfully engage and enhanced therapy experience | 15 (78.9%) | 13 (81.3%) | 2 (66.7%) |
|  | ***Significant impact of therapeutic alliance****:* Strong therapeutic relationship overcame potential barriers and facilitated engagement and outcomes | 16 (84.2%) | 14 (87.5%) | 2 (66.7%) |
| Individual approach and experience: Participants’ attitudes and approaches across therapy impacted engagement, experience and outcomes | ***Open-minded attitudes despite initial doubts****:* Central aspect of initial engagement related to approaching therapy with a willingness to try | 18 (94.7%) | 15 (93.8%) | 3 (100%) |
|  | ***Determination facilitated engagement and outcomes****:* Resilience and perseverance were important for keeping going with therapy and getting the most out of the experience | 17 (89.5%) | 16 (100%) | 1 (33.3%) |
|  | ***Profound emotional experience****:* The depth of emotional experience, encompassing demands and valued experiences | 17 (89.5%) | 14 (87.5%) | 3 (100%) |
|  | ***Offered novel approach to tackle voices****:* Working dialogically with verbatim voice content opened up new possibilities to engage differently with voices | 12 (63.2%) | 11 (68.8%) | 1 (33.3%) |
| Barriers to engagement and outcomes: Aspects of experience which inhibited meaningful engagement and positive outcomes. | ***Emotional challenges with avatar****:* Difficulties working with voices via the avatar | 10 (52.6%) | 7 (43.8%) | 3 (100%) |
|  | ***Not the right approach for the individual at that time****:* Considering individual factors, the nature of voices and stage in recovery, AVATAR therapy may not be engaging and/or effective for all | 8 (42.1%) | 5 (31.3%) | 3 (100%) |
|  | ***Difficulties translating changes to voices****:* Barriers to translating improvements from avatar to voices | 6 (31.6%) | 6 (37.5%) | 0 (0%) |
