# Supplementary material for: “It Felt Good to Be Able to Say That Out Loud”—Therapeutic Alliance and Processes in AVATAR Therapy for People Who Hear Distressing Voices: Peer-Led Qualitative Study
Source: JMIR Ment Health. 2026 Jan 28;13:e77566. doi: 10.2196/77566 (PMC12895157; doi:10.2196/77566)
Supplement: Multimedia Appendix 7 [file mental_v13i1e77566_app7.docx]

**Supplementary material 7: Illustrative quotes by subtheme**

| Theme and subtheme | Participant (pseudonym) | Quote |
| --- | --- | --- |
| **Shift in relationship with avatar and, consequently, voices** | | |
| Initial challenges adjusting to avatar | Caleb (AV-EXT, completed therapy) | ‘I didn’t believe that the voice could become real and that it had a face now […] so I didn’t really believe that it could happen, but it did’ |
|  | Grace (AV-EXT, dropped out of therapy) | ‘His face was realistic, it was just like the devil and that was scary but as if it was real rather than just hearing the voice.’ |
|  | Asim (AV-BRF, completed therapy) | ‘I’m so used to just hearing it in my head that actually hearing it out loud, it was a bit uncomfortable’ |
|  | Charlotte (AV-BRF, completed therapy) | ‘Because I had designed a face like a character, my fear was that if I had another relapse and this figure is going to start showing, instead of it just being an audio hallucination, it’s going to be a visual hallucination. And that’s what was terrifying me the most’ |
|  | Gabriel (AV-EXT, completed therapy) | ‘I think it's completely impossible to actually design an avatar to replicate exactly what is going on’ |
|  | Stephen (AV-BRF, completed therapy) | *‘*It would be easier if people could really resonate with that avatar, and I got a lot out of it, but I feel like there's a lot of people who may be put off by it or not see the benefit of it because it's not as refined as it could be’ |
| Collaborative efforts facilitated meaningful connection to avatar | Paula (AV-BRF, completed therapy) | ‘In the initial state, it wasn’t anything close to that I was familiar with, but I have to work with it to liken it to what I was going through’ |
|  | William (AV-EXT, completed therapy) | ‘He was quite a good actor [laughs], so he was able to put things together in terms of feelings and emotions in around it’ |
|  | Gabriel (AV-EXT, completed therapy) | ‘The more it went on, the more useful it became because I was, it felt like I was more actually addressing the voices in my head because they were becoming more like the avatar throughout’ |
| With therapist support, participants felt empowered to stand up to avatar | William (AV-EXT, completed therapy) | ‘That’s the other strength of it […] hopefully looking at somebody or something you can identify with the voice […] and then once you’re able to answer back, when you really want to, it gives it more punch’ |
|  | Stephen (AV-BRF, completed therapy) | ‘It was really down to the help of the therapist to really shift my mind outside of those conversations and ask me ‘well, why did you answer like this?’ and like, ‘do you feel like this?’ or ‘are you aware of X, Y and Z?’ so it was, it was very helpful for me to have that self-reflection after the conversation’ |
|  | Grace (AV-EXT, dropped out of therapy) | ‘I could actually see him and talk to him, and getting a wee bit of power rather than being power- overpowering me’ |
|  | Joshua (AV-BRF, completed therapy) | ‘I just felt like I was kind of unravelling with it a little bit, like unravelling my brain with it’ |
| Positive shift with voices | William (AV-EXT, completed therapy) | ‘It takes me from a place where I’d get very emotional and very angry to [pause] I think it doesn’t bother me, it just doesn’t bother me anymore’ |
|  | Charlotte (AV-BRF, completed therapy) | ‘Just taking back control and not believing everything that he says’ |
|  | Matthew (AV-EXT, completed therapy) | ‘They didn’t go away, but it gives me a better understanding about why they were there, and it gave me a better understanding about psychosis’ |
|  | Asim (AV-BRF, completed therapy) | ‘Helped me understand the voices a bit more and help me heal with what they say’ |
|  | Matthew (AV-EXT, completed therapy) | ‘I’ve finished with the AVATAR now about seven or eight months and I’m still finding the skills and the understanding’ |
|  | Grace (AV-EXT, dropped out of therapy) | ‘It helped me deal with things a bit better and try get on with my life and be able to control things that’s going on in my life’ |
| **Crucial role of person-centred therapist** | | |
| Felt safe, supported and understood | Elif (AV-BRF, completed therapy) | Sometimes my English is broken and then [I] can’t explain everything […] directly, and then I’m scared that […] she doesn’t understand me’ |
|  | Gabriel (AV-EXT, completed therapy) | ‘[Therapist]’s completely separate to every other part of my life, so it’s quite a nice little bubble to just say whatever I wanted to say’ |
|  | Joshua (AV-BRF, completed therapy) | ‘I could tell [therapist] was genuine, genuinely like invested in helping me’ |
|  | Paula (AV-BRF, completed therapy) | ‘She stayed professional and besides being professional, she made me feel comfortable. And that was very important to me because I was letting her into my space and letting her into my head to know what I was feeling’ |
|  | Zahid (AV-BRF, completed therapy) | ‘I was explained step by step through the whole process as as things were happening […] I fully understood what was happening all the way’ |
| Person-centred flexibility | Matthew (AV-EXT, completed therapy) | ‘They were very, very, very flexible with me, which I found dead helpful’ |
|  | Paula (AV-BRF, completed therapy) | ‘Every time I would have like an emotional breakdown, my therapist […] wasn’t hounding me, she would let me breathe through that breakdown and let me, if I had to express myself, I did. If I wanted to stop the session and call it a day, I was able to do all that. So all those things, it just happened natural for me’ |
|  | Joshua (AV-BRF, completed therapy) | ‘The fact that he would take the time, like to really like unravel with me was really helpful’ |
|  | Paula (AV-BRF, completed therapy) | ‘Especially the fact that they made it possible for me to attend my sessions and um getting me back home safe, that was very very helpful’ |
| Significant impact of therapeutic alliance | Matthew (AV-EXT, completed therapy) | ‘I felt we connected; we made a connection’ |
|  | Asim (AV-BRF, completed therapy) | ‘I would say I put more trust into the therapist, so I was willing to give what I learned in the therapy at home to my actual voices’ |
|  | Caleb (AV-BRF, completed therapy) | ‘I was able to like separate, separate them, and I understood that that was that person and that was that person’ |
|  | Caleb (AV-EXT, completed therapy) | ‘When I hear it now, I just sit back and think what like what my therapist would say’ |
|  | Zahid (AV-BRF, completed therapy) | ‘What I benefitted from it from the most was just coming in and seeing someone and talking about it’ |
| **Individual approach and experience** | | |
| Open-minded attitudes despite initial doubts | Caleb (AV-EXT, completed therapy) | ‘I was a bit like dubious, because I didn't know what it would entail’ |
|  | Charlotte (AV-BRF, completed therapy) | ‘When I read up on it, to me, I was like, ‘it's just going to be silly, like I just don't understand how this could help somebody’. So I I would say I was quite sceptical to begin with’ |
|  | Caleb (AV-EXT, completed therapy) | ‘I kind of went in open-minded’ |
|  | Matthew (AV-EXT, completed therapy) | ‘I was quite excited by it to be quite honest. I felt as though […] it's clearly identified something in me that was looking out for a therapeutic experience’ |
|  | Henry (AV-BRF, completed therapy) | ‘I thought it was something to do that would lead somewhere’ |
|  | Stephen (AV-BRF, completed therapy) | ‘I wasn't really expecting to get much from it at the very least I thought it would start a conversation that I would be able to then continue in other therapies or with myself going forward’ |
|  | Charlotte (AV-BRF, completed therapy) | ‘Family and friends that actually encouraged me to do it because they said ‘if you don't try, you would never know’.’ |
|  | Stephen (AV-BRF, completed therapy) | ‘The psychologist that was leading my treatment at [mental health service] was a big influence […] when he explained it to me, I definitely trusted his judgement’ |
|  | Joshua (AV-BRF, completed therapy) | ‘I mostly like wanted it myself. I wanted to get better’ |
|  | Paula (AV-BRF, completed therapy) | ‘You never know until you try’ |
|  | Ishan (AV-EXT, completed therapy) | ‘Be open minded um and think about it as a tool to assist you in your recovery rather than thinking too much about ‘it's the voices coming at me and I don't want to to hear more of the voices anymore’.’ |
| Determination facilitated engagement and outcomes | Joshua (AV-BRF, completed therapy) | ‘Motivation to get better. […] What I worry about constantly is being a burden to other people, and if I want to not be a burden, then I've got to take every resource and every chance I can to get better, I can't just sit around and mope’ |
|  | Charlotte (AV-BRF, completed therapy) | ‘Just trying to see if I can better myself each week, slight improvements each week and then looking at the positives, so what I did achieve on these sessions’ |
|  | Ishan (AV-EXT, completed therapy) | ‘Comparing my previous self to where I was at next session basically, and I think it was just every session I was going, […] I could see the noticeable difference from my previous session’ |
|  | Angus (AV-EXT, completed therapy) | ‘Opening up. You basically had to open up, otherwise there’s no point doing it’ |
|  | Charlotte (AV-BRF, completed therapy) | ‘I'd probably say it's going to be difficult, but it does get easier over time. If you try it, if you stick at it’ |
|  | Grace (AV-EXT, dropped out of therapy) | ‘It's a process. You just have to keep going and keep going and giving it all you can’ |
|  | Charlotte (AV-BRF, completed therapy) | ‘Don't try and chuck it after the first session like I tried to do’ |
|  | Stephen (AV-BRF, completed therapy) | ‘To be honest with you like it is what you make of it, I feel like you could have given me a robot with a robotic voice, and it still would have had a profound impact on me if I was willing to do the work’ |
|  | Mai Su (AV-BRF, completed therapy) | ‘I think my six sessions was quite hard because there was quite long gaps between them […] I was missing appointments and so it was quite it was quite drawn out over the months’ |
| Profound emotional experience | Stephen (AV-BRF, completed therapy) | ‘I can't even feel comfortable to say how much depth I went through in that, but it was very raw’ |
|  | Alexander (AV-EXT, dropped out of therapy) | ‘I don't have words for it, to be honest’ |
|  | Matthew (AV-EXT, completed therapy) | ‘I think something in me changed’ |
|  | Paula (AV-BRF, completed therapy) | ‘Those little moments, they were lifechanging’ |
|  | Caleb (AV-EXT, completed therapy) | ‘I did get angry once, I punched a wall’ |
|  | Charlotte (AV-BRF, completed therapy) | ‘Drained is the word that I would use for myself, emotionally drained’ |
|  | Mai Su (AV-BRF, completed therapy) | ‘I got like a bit anxious over the voice talking back to me on the screen and my heart would be racing a bit, but I didn't get upset, I didn't cry or anything, so it was a lot better [than CBT]’ |
|  | Ishan (AV-EXT, completed therapy) | ‘Ready to leave, sort of ready to go to the next stage’ |
|  | Charlotte (AV-BRF, completed therapy) | ‘I wish we did have more sessions because as I say a few of the sessions to begin with were really, really tough. So […] I never really got to speak much because I was too busy crying [laughs] I just felt if it was a wee bit more sessions I think for me would have brought a bit more closure’ |
| Offered novel approach to tackle voices | Joshua (AV-BRF, completed therapy) | ‘That was all that was intriguing, it was like different […] I've received therapy in the in the past but nothing like this’ |
|  | Asim (AV-BRF, completed therapy) | ‘It was interesting to hear about because he said it was uh kind of new and [pause] it was an opportunity to see if it would help with the voices’ |
|  | Joshua (AV-BRF, completed therapy) | ‘I wouldn't have thought of doing that without the therapy’ |
|  | Asim (AV-BRF, completed therapy) | ‘Before the avatar, I would never talk back to it, so it was the first time for me’ |
|  | Gabriel (AV-EXT, completed therapy) | ‘I started having a dialogue with the voice about why that was happening, which I don't think would ever have happened separately, then it was almost like I felt like I was able to influence the voices in my head so that we could then talk about why what was going on was going on’ |
| **Barriers to engagement and outcomes** | | |
| Emotional challenges with avatar | Alexander (AV-EXT, dropped out of therapy) | ‘The hammer came down like ‘No. […] I'm not pussyfooting around this anymore’ for the lack of a better terminology, I was like ‘No, fuck this. None of this is beating me now, I'm not going to feel weak anymore’.’ |
| Not the right approach for the individual at that time | James (AV-EXT, dropped out of therapy) | ‘It was a bit misguided, to be honest’ |
|  | Stephen (AV-BRF, completed therapy) | ‘You really lend yourself to the experience […] I would definitely question if somebody is ready to do that and willing to fully commit to the process’ |
| Difficulties translating changes to voices | Asim (AV-BRF, completed therapy) | ‘I didn’t know what to do when it was angry, so I told that to my support worker, who contacted [therapist] and [therapist] um helped me out’ |
|  | Gabriel (AV-EXT, completed therapy) | ‘I was also very reluctant to do so, um, but yeah, there's two sides of the coin, I didn't like humanising them particularly’ |
